# Wrinkle‐Adaptive Kirigami Wearables With Anisotropic Deformability for Sleep EEG Monitoring

**DOI:** 10.1002/advs.202600035

**Published:** 2026-03-20

**Authors:** Jungmin Kim, Ka Ram Kim, Byeongjun Lee, Cheoljeong Park, Seong J. Cho, Woon‐Hong Yeo

**Affiliations:** ^1^ Department of Mechanical Engineering Chungnam National University Daejeon Republic of Korea; ^2^ Wearable Intelligent Systems and Healthcare Center (WISH Center) At the Institute for Matter and Systems Georgia Institute of Technology Atlanta Georgia USA; ^3^ Korea KIAT‐Georgia Tech Semiconductor Electronics Center At the Institute for Matter and Systems Georgia Institute of Technology Atlanta Georgia USA; ^4^ George W. Woodruff School of Mechanical Engineering Georgia Institute of Technology Atlanta Georgia USA; ^5^ Wallace H. Coulter Department of Biomedical Engineering Georgia Institute of Technology Emory University School of Medicine Atlanta Georgia USA; ^6^ Parker H. Petit Institute For Bioengineering and Biosciences Georgia Institute of Technology Atlanta Georgia USA

**Keywords:** anisotropic deformability, EEG monitoring, kirigami structure, sleep, wearable patch, wrinkle adaptive

## Abstract

Wearable electroencephalography (EEG) devices offer a promising solution for continuous brain monitoring outside laboratory settings. However, maintaining stable signal quality over extended periods remains challenging, as skin‐mounted EEG systems are prone to contact‐induced noise caused by skin deformation and body motion. Here, we present a wrinkle‐adaptive kirigami structure and a soft, wearable EEG patch engineered to match subject‐specific forehead wrinkle patterns, thereby stabilizing the electrode‐skin interface. Our two‐step kirigami architecture integrates global conformability with localized strain accommodation, delivering anisotropic deformability and maintaining mechanical stability during facial motion. Leveraging an automated image‐based workflow, we enable scalable, individualized generation of kirigami patterns. When integrated into a soft, wireless wearable system, the personalized patch delivers consistently enhanced signal‐to‐noise ratios across multiple EEG frequency bands, even under diverse motion conditions in at‐home sleep settings. This technology demonstrates broad applicability for sleep EEG monitoring and underscores the potential of morphology‐aware structural design for next‐generation wearable EEG devices.

## Graphical abstract

This article introduces a wrinkle‐adaptive, kirigami‐structured wearable EEG patch that personalizes electrode‐skin conformity to stabilize the interface and enable wireless, high‐quality sleep monitoring.

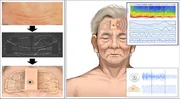

## 1 Introduction

Electroencephalography (EEG) provides real‐time access to brain dynamics and has been widely adopted in applications ranging from neurological diagnosis [1, 2, 3, 4] and cognitive state monitoring to brain‐computer interfaces (BCIs) [5, 6, 7, 8, 9, 10, 11, 12], human–computer interaction (HCI) [2, 13, 14, 15, 16], mental health assessment [17, 18, 19], and long‐term neurophysiological monitoring [3, 13, 20]. Its high temporal resolution and compatibility with lightweight electronics make EEG particularly attractive for long‐term and ambulatory applications [10, 12, 21, 22]. However, achieving stable, high‐fidelity EEG acquisition in unconstrained environments remains challenging due to motion artifacts and unstable electrode–skin interfaces. Conventional scalp EEG systems based on fixed electrode configurations are especially vulnerable to signal degradation during prolonged use, as hair‐covered regions and head motion introduce contact instability and noise. To address these limitations, prior efforts have explored invasive or semi‐invasive electrodes [10, 23, 24, 25, 26, 27], bulky whole‐head systems [20, 28, 29, 30, 31, 32, 33], and algorithm‐based signal denoising. While these approaches can partially mitigate motion artifacts, they remain limited by invasiveness, poor wearability, or strong dependence on data processing, restricting their suitability for long‐term, real‐world monitoring. Against this backdrop, increasing attention has been directed toward forehead‐based EEG monitoring, which benefits from minimal hair interference and improved wearability [9, 19, 20, 32, 33, 34, 35, 36]. Nevertheless, the forehead presents a distinct mechanical challenge. As a highly dynamic facial region, it undergoes frequent skin deformation during facial expression, posture changes, and sleep‐related motion [19, 31, 37, 38, 39]. Moreover, forehead skin exhibits pronounced, age‐dependent wrinkle structures that vary significantly across individuals. Given that the majority of clinical EEG applications target older populations, a design strategy that merely seeks to circumvent these wrinkles is impractical. Therefore, it is essential to implement a methodology that configures the patch structure based on quantitative analysis of individual wrinkle patterns. Most prior studies addressing forehead EEG noise have primarily relied on algorithmic strategies such as preprocessing, artifact rejection, or machine‐learning‐based classification [37, 40]. In parallel with algorithm‐driven approaches, recent advances in wearable bioelectronics have focused on mechanically compliant epidermal systems to improve signal stability. High‐performance electronic tattoos with serpentine or ultrathin architectures have demonstrated enhanced conformal contact and long‐term electrophysiological monitoring capabilities, while integrated perception–memory platforms combining stretchable sensors with memristive devices have enabled advanced human–machine interaction and intelligent signal processing [41, 42, 43]. These systems represent significant progress in stretchable and multifunctional bioelectronics. However, most reported strategies primarily enhance global stretchability through intrinsic material softness or serpentine interconnect geometries, without explicitly engineering structural strain redistribution at heterogeneous skin interfaces. In particular, geometry‐programmed and subject‐specific design strategies that account for anisotropic, wrinkle‐rich facial regions remain largely unexplored.

Here, we present a personalized kirigami‐structured wearable EEG patch that structurally suppresses motion‐induced noise by stabilizing the electrode–skin interface in forehead EEG monitoring. The device design leverages the anisotropic deformability of kirigami architectures to conform to horizontally aligned, age‐dependent forehead wrinkle patterns while preserving mechanical integrity during facial motion. A kirigami strategy was implemented, comprising a primary kirigami pattern that enhances global conformability and prevents electrode delamination across the forehead, and locally refined kirigami features that accommodate wrinkle‐induced strain at the skin interface [44, 45]. Mechanical optimization based on modulus matching and peeling behavior enabled close alignment between the patch's effective mechanical response and that of forehead skin. An automated image‐based workflow further allows scalable generation of subject‐specific kirigami patterns directly from individual forehead images. When integrated into a compact, wireless wearable system, the personalized kirigami patch demonstrates a substantial improvement in EEG signal quality across multiple frequency bands and motion conditions during at‐home sleep.

## 2 Result and Discussion

### 2.1 Fabrication and Integration of a Soft, Personalized Kirigami EEG Patch

**FIGURE 1:**
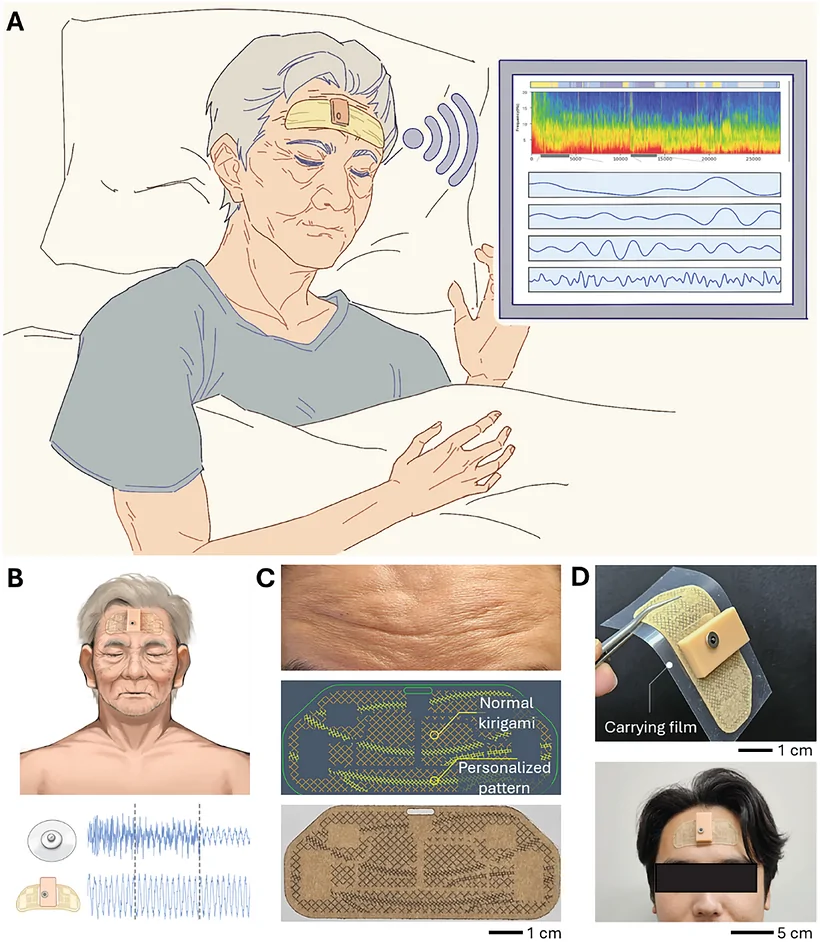
Overview of a personalized kirigami‐structured forehead patch for sleep EEG monitoring. (A) Illustration of the wearable EEG patch designed for forehead application, highlighting the soft, skin‐conformal architecture and dry electrode configuration optimized for unobtrusive electrophysiological monitoring. (B) Illustration of a subject wearing the compact and lightweight EEG monitoring system, emphasizing its low‐profile form factor and enhanced SNR compared to conventional gel‐based electrodes. (C) Schematic of data flow for personalized kirigami feature design, followed by fabrication of the substrate. (D) Optical image of the fully integrated EEG system mounted on a carrying film and a photograph of the system worn on the forehead, demonstrating its conformal attachment and system‐level integration.

Our system is a fully encapsulated, forehead‐mounted wearable EEG platform that enables continuous, wireless acquisition of multichannel EEG signals during long‐term use, including sleep monitoring, as illustrated in Figure 1A. The soft, kirigami‐patterned wearable patch maintains conformal electrode‐skin contact during facial expressions and head motion, addressing the dominant sources of motion artifacts in wearable EEG recordings compared to commercial gel electrodes (Figure 1B). Personalized kirigami patches are generated through an automated image‐processing workflow that extracts individual forehead wrinkle features and translates them into customized kirigami designs, enabling adaptation to inter‐subject skin morphology (Figure 1C). Figure 1D shows that the kirigami patch exhibits high flexibility and low bending stiffness, as demonstrated on a thin carrying film. It maintains a low‐profile, unobtrusive form factor when worn on the forehead. These features demonstrate how the system‐level integration of full encapsulation, automated personalization, and mechanically adaptive kirigami structures enables reliable EEG acquisition across daily activities and extended sleep monitoring.

**FIGURE 2:**
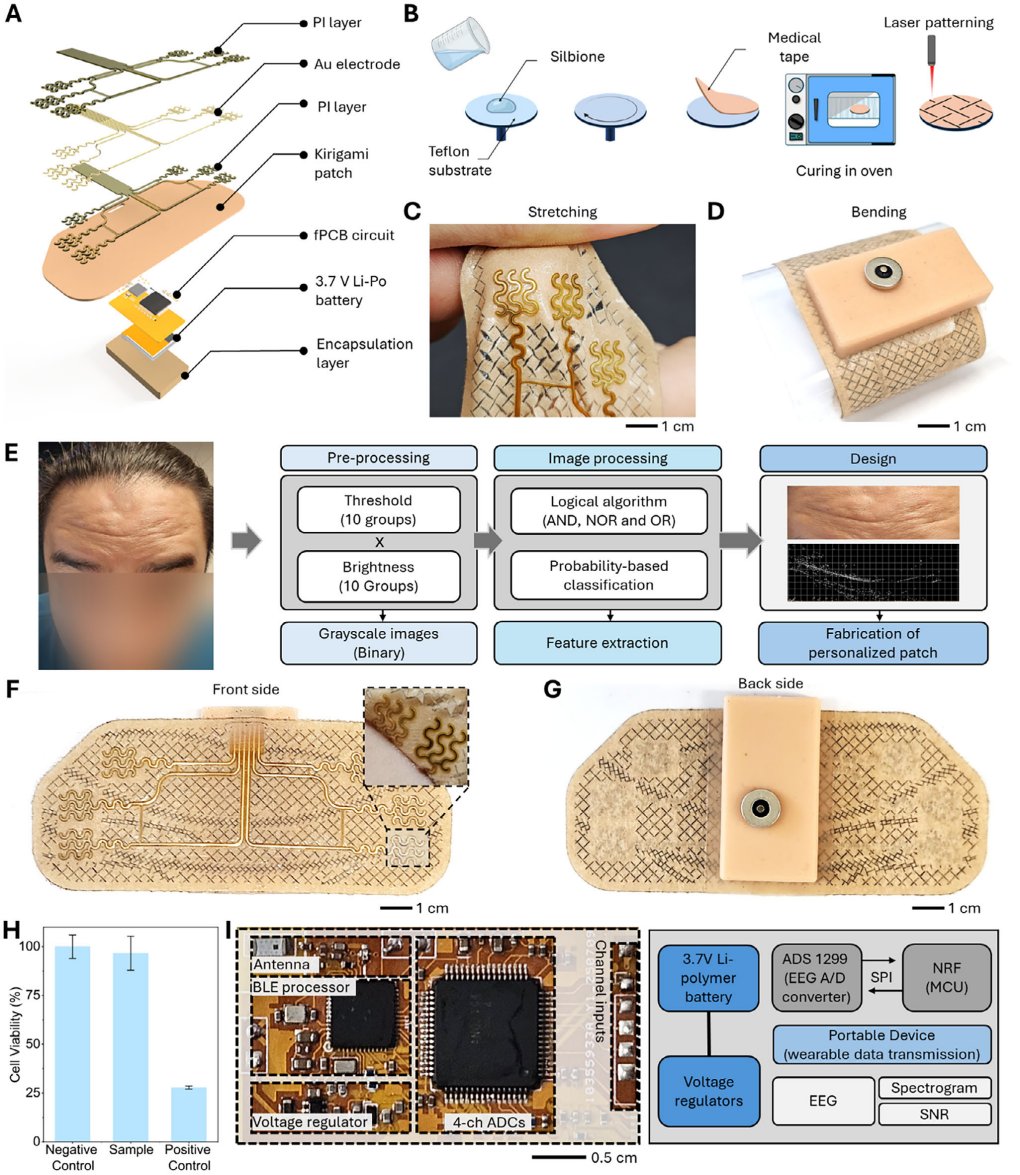
Design, architecture, and fabrication process for a wearable sleep device. (A) Exploded schematic illustration of the kirigami‐based wearable EEG patch (B). Fabrication process of the kirigami‐patterned membrane substrate with an adhesive layer, followed by laser processing. (C) Photo of sleep patch showing stretchability. (D) Photo of a sleep patch showing conformal contact on a bent surface. (E) Workflow for wrinkle image acquisition, automated feature extraction, and algorithm‐assisted fabrication of a personalized kirigami patch. (F) Integrated devices with an electrode attached (inlet: electrode‐skin interface). (G) Integrated circuits with a battery charger, fully encapsulated by a silicone membrane. (H) Cell viability in the sleep patch demonstrates high biocompatibility. (I) An image of a flexible printed circuit board and a diagram showing the sequence for monitoring an EEG signal via Bluetooth‐based wireless data transmission to a portable device, followed by data processing.

Figure 2 summarizes the fabrication workflow and system‐level realization of a soft, wearable EEG device, highlighting the structural and mechanical characteristics that enable long‐term forehead monitoring. Figure 2A shows the fully integrated portable platform, consisting of a forehead‐mounted EEG sensor array (PI/Au/Pi stacked layers) laminated onto a kirigami‐patterned medical fabric substrate with a soft silicone adhesive layer (Silbione, Elkem), and a flexible PCB powered by a 3.7 V Li‐polymer battery. The entire devices are fully encapsulated using a silicone elastomer membrane (Ecoflex 00–30, Smooth‐On), formed by mold casting with a 3D‐printed mold, providing mechanical stability and reliable operation during extended wear. The fabrication procedures for the medical fabric‐adhesive composite and the kirigami structures are depicted in Figure 2B. A thin, compliant silicone adhesive layer is prepared on a poly(tetrafluoroethylene) (PTFE) substrate by spin coating and thermal curing, followed by lamination of an elastic nonwoven polyurethane medical fabric (9970T, 3 M) to form a conformal adhesion interface suitable for skin contact. High precision kirigami perforations are subsequently introduced using nanosecond laser machining (PhotoLaser U4, LPKF), patterning through both the adhesive layer and the fabric substrate. After kirigami patterning, the dry electrodes are assembled, integrated with the flexible PCB, and finally encapsulated with Ecoflex 00–30 using a 3D‐printed SLA mold. Figure 2C,D highlights the skin‐contacting side of the device, where the kirigami‐structured region exhibits high stretchability while suppressing interfacial delamination. The device also demonstrates pronounced flexibility when applied to curved surfaces with a curvature of 0.4mm^−1^, supporting conformal lamination on the forehead during deformation. The workflow for designing personalized kirigami structures is presented in Figure 2E. Individual forehead images are acquired and processed through an automated image‐processing pipeline that includes feature extraction and probability‐based classification of wrinkle patterns. The extracted wrinkle features are then overlaid with kirigami designs to generate subject‐specific designs. All steps are performed automatically using ImageJ macros and AutoCAD software, enabling scalable fabrication of personalized patches without manual intervention (see Experimental Section 4.2 for more details). Figure 2F,G shows the fully integrated and encapsulated personalized kirigami patch, emphasizing its compact, low‐profile form factor compared to conventional wearable sleep monitors. The kirigami‐enabled soft patch architecture, comprising a thin, low‐modulus adhesive layer, a biocompatible fabric substrate, and a silicone encapsulation, promotes intimate and stable skin lamination, which is critical for reducing motion‐induced contact disturbances during long‐term recordings. To assess the biocompatibility of the assembled patch materials, cytotoxicity was assessed using L929 cells. Following extraction, the samples were immersed in cell culture media for 24 h, and the extracted media were then applied to healthy L929 cells and cultured for 72 h. As shown in Figure 2H, the MTS assay revealed negligible differences in cell viability between the negative control and our sample (96.6 %). Consequently, these results demonstrate that the fabricated patch is highly biocompatible. Finally, Figure 2I presents the architecture of the flexible PCB and the signal‐processing and transmission pathways. The system includes a multichannel analog front end and ADC (ADS1299), power regulation stages, and a Bluetooth Low Energy microcontroller (nRF52832) connected to a 2.4 GHz antenna. Acquired EEG signals are digitized and transmitted wirelessly to portable devices (e.g., smartphones or tablets) for real‐time visualization and monitoring. A detailed summary of the IC components for the EEG patch is depicted in Figure S1. Overall, the fabrication‐integrated soft device design, kirigami‐enabled mechanical compliance, full encapsulation, and validated biocompatibility collectively support a wearable EEG platform suitable for reliable forehead EEG acquisition during long‐term, unconstrained use.

### 2.2 Wrinkle‐Adaptive Mechanical Behavior of Kirigami Structures

**FIGURE 3:**
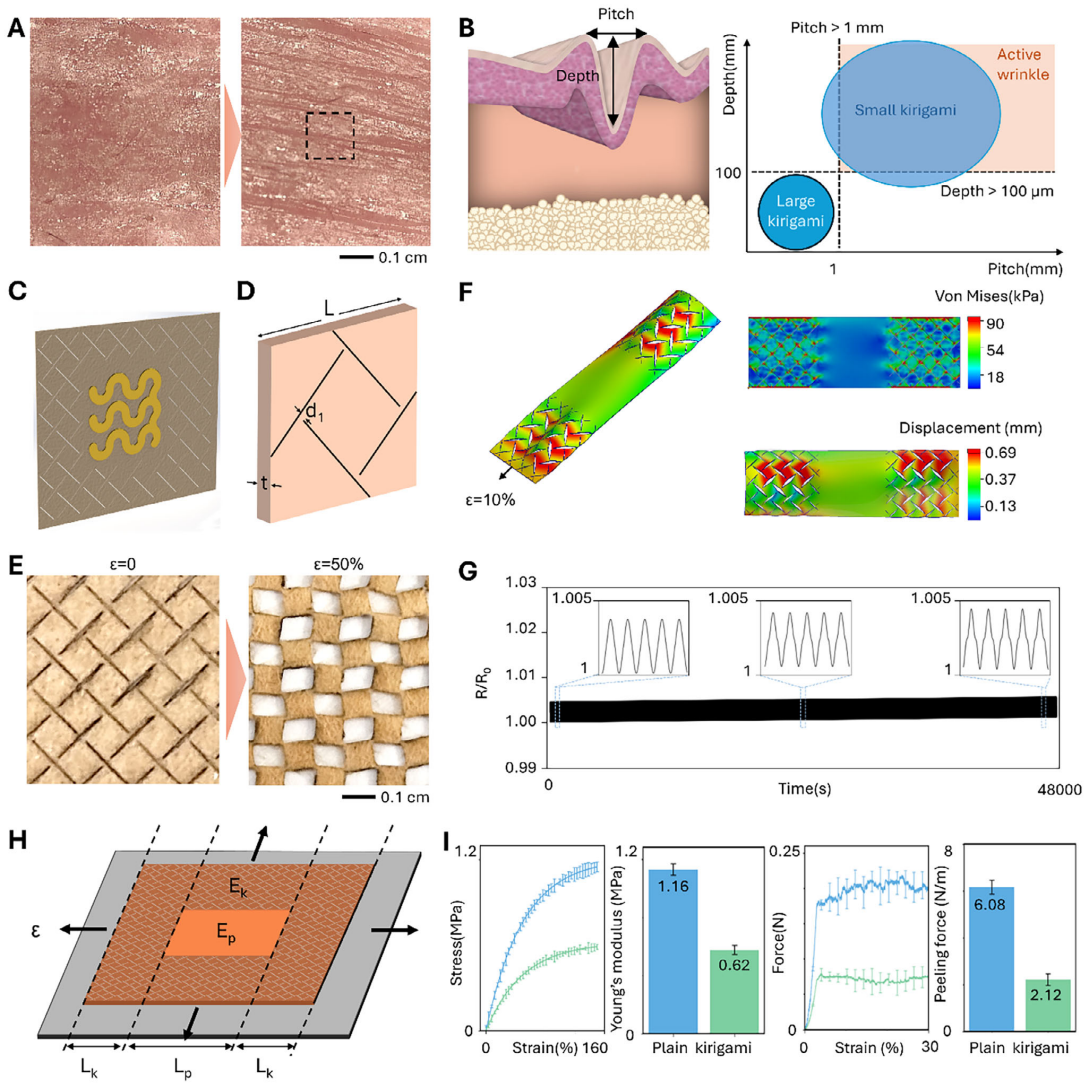
Theoretical study and mechanical characterization of kirigami structure. (A) Optical image of forehead skin before and after frowning. (B) Illustration of wrinkle structure and effective regions of both depth and pitch according to the size of the kirigami. (C) An illustration of an electrode on kirigami structured membrane substrate. (D) Detailed scale of a single bi‐axial kirigami pattern unit. (E) Deformation results show a kirigami pattern with 50 % biaxial strain, with fully rotated (90°) segments and stretched segments. (F) FEM simulation of kirigami patterns: Stress and displacement, respectively. (G) Change in electrical resistance of a fully encapsulated personalized kirigami patch throughout a cyclic stretching test (2000 cycles at 10 % strain), demonstrating reliable resistance changes. (H) Schematic illustration of the strain‐partitioning mechanism in the personalized kirigami patch. (I) Young's modulus and peeling strength of plain patches and kirigami patches.

EEG signals acquired from the forehead are highly susceptible to motion‐induced artifacts arising from facial expressions, eye movements, and head motion [46]. These artifacts primarily originate from dynamic changes at the electrode–skin interface, where variations in contact state and skin–electrode impedance directly degrade signal quality [46, 47, 48]. Although soft adhesive layers are commonly employed to improve conformal contact, dry electrodes laminated onto fabric substrates still experience contact instability under repeated or large deformation. Mechanical mismatch between the relatively stiff electrode components and the soft adhesive–fabric composite can induce interfacial slippage or partial delamination during motion, leading to transient impedance fluctuations and, in some cases, long‐term signal degradation [49, 50]. To achieve reliable EEG acquisition under dynamic conditions, the electrode–skin interface must simultaneously satisfy two competing requirements: intimate conformal contact and mechanical decoupling from externally applied strain. When the patch deforms with the skin without accommodating local strain concentration, externally induced deformation is directly transmitted to the electrode region, destabilizing the interface. To address this challenge, a kirigami structure was introduced into the substrate surrounding the dry electrodes. The kirigami pattern enables controlled anisotropic deformation of the substrate, redistributing applied strain away from the electrode region while preserving conformal lamination to the skin. As a result, the electrode maintains consistent contact during various motions, improving signal stability, electrode durability, and reusability, as shown in Figure 3. Figure 3A shows the representative optical images of human forehead skin, revealing pronounced, anisotropic wrinkle patterns that vary in depth and pitch across individuals. These wrinkle patterns form a heterogeneous and dynamically deforming surface rather than a planar interface. Figure 3B schematically shows a cross‐sectional view of the wrinkle geometry, along with the conformal coverage regions achieved by kirigami structures of different feature sizes. In regions where wrinkles exhibit large depth (typically exceeding ∼100 µm) and millimeter‐scale pitch, kirigami structures with relatively large unit features tend to undergo localized detachment or partial delamination due to insufficient bending compliance. In contrast, smaller‐scale kirigami structures exhibit a reduced effective modulus, allowing them to deform cooperatively with skin motion and accommodate wrinkle‐induced curvature without interfacial separation. This compliance‐dominated behavior provides a mechanical rationale for tailoring the size of kirigami features to individual wrinkle morphologies in personalized patch designs. Based on this morphological consideration, kirigami structures were introduced into the adhesive–fabric substrate surrounding the dry electrodes to mechanically isolate the electrode region from wrinkle‐induced deformation. Figure 3C shows the fabric patch and adhesive layer perforated with diagonal kirigami patterns surrounding the gold dry electrodes on the skin‐contacting side of the patch. The electrode regions were intentionally preserved as structure‐free zones to maintain uniform contact pressure and electrical stability at the electrode–skin interface, while the surrounding kirigami patterns provide mechanical strain isolation [51, 52]. Two features of kirigami configurations were implemented: a large‐scale kirigami pattern applied across the entire patch area to provide global stretchability, and a small‐scale kirigami pattern introduced locally in regions corresponding to pronounced forehead wrinkles to enhance conformal contact at the electrode–skin interface. The corresponding unit cell geometry is illustrated in Figure 3D, where the kirigami pattern consists of a tilted square lattice defined by the in‐plane unit width *L*, gap size *d_1_*, and composite thickness *t*. In this geometry, each kirigami unit is composed of slanted slit elements with a length of 3.04 mm oriented at 45° relative to the principal axes. Based on the geometric projection of the slits, the characteristic in‐plane unit width of the large‐scale kirigami design is approximately *L* ≈ 4.4 mm, with an inter‐slit gap size *d_1_* of 0.175 mm. The small‐scale kirigami design was generated by uniformly reducing the in‐plane dimensions (L and *d_1_*) by a factor of two while maintaining an identical composite thickness *t*. By keeping the thickness constant across all designs, differences in mechanical behavior arise primarily from in‐plane geometric scaling rather than changes in material thickness. When external tensile strain is applied, these diagonal kirigami structures rotate at the cut corners, effectively releasing the deformation near the electrodes. As shown in Figure 3E, under a tensile strain of 50 %, the kirigami units rotate and open at the hinges, enabling large global deformation while minimizing local strain transfer to the electrode region. Finite element analysis further elucidates the strain‐decoupling mechanism. Figure 3F presents simulated stress and displacement distributions for kirigami‐patterned patches subjected to 10 % uniaxial stretching. The results show that stress is concentrated within the kirigami units and hinge regions, whereas negligible deformation is transmitted to the central area where the electrodes are attached. This localized strain accommodation demonstrates that the kirigami architecture effectively shields the electrode‐skin interface from externally applied deformation, consistent with the wrinkle‐adaptive design rationale. To evaluate mechanical durability under prolonged dynamic loading, the electrical reliability of fully encapsulated kirigami patches was assessed through cyclic tensile testing. As shown in Figure 3G, the patches were repeatedly stretched and relaxed at 120 mm/min for 2000 cycles of 20 % tensile stretching, corresponding to more than 13 h of continuous mechanical loading, followed by the setup depicted in Figure S2. Throughout the cycling test, electrical resistance remained within 0.5 % of its initial value, exhibiting negligible variation. Resistance traces recorded at the early (cycles 0–5), intermediate (cycles 990–995), and late (cycles 1990–1995) stages further confirm the long‐term mechanical and electrical stability of the kirigami structure. Together with the cyclic tensile endurance (Figure 3G), the stable time‐ and moisture‐dependent peeling performance (Figure S3) confirms the long‐term mechanical and interfacial reliability of the kirigami patch for extended wearable EEG monitoring. In addition to mechanical and interfacial stability, long‐term skin compatibility was evaluated through extended forehead wear tests. Representative post‐removal images after 8 h of simultaneous attachment of a conventional gel electrode and the fully encapsulated personalized kirigami patch are shown in Figure S3. Visible erythema was observed at the gel electrode site, whereas only minimal and transient skin impressions were observed at the kirigami patch site, indicating improved wear comfort during prolonged monitoring. The underlying mechanical mechanism is schematically illustrated in Figure 3H. When a global in‐plane strain ε_0_ is applied to the patch, the deformation is non‐uniformly distributed between the kirigami‐structured region and the electrode‐adjacent plain region. Due to the reduced effective modulus of the kirigami lattice (E_k_ <E_p_), the majority of the applied strain is accommodated within the kirigami region over the length L_k_ through rotational and bending deformation of the cut units. In contrast, the plain region with a higher modulus E_p_ experiences a significantly smaller effective strain ε_p_ over its characteristic length L_p_. As a result, the local strain transmitted to the electrode‐adjacent region is strongly suppressed (ε_p_< ε_0_), effectively mechanically isolating the electrode–skin interface from externally applied deformation. This strain‐partitioning mechanism prevents interfacial slippage or delamination during skin motion while preserving sufficient normal adhesion for out‐of‐plane compliance. Figure 3H provides a conceptual framework for strain partitioning, which is quantitatively validated by the experimental modulus and peeling measurements. Quantitative mechanical characterization further highlights the impact of kirigami patterning on patch compliance and interfacial behavior. Figure 3I compares the tensile and peeling properties of plain and kirigami‐patterned patches, and the detailed test setups are shown in Figures S3 and S4. Plain patches exhibit a higher Young's modulus of 1.16 MPa and a higher peeling force of 6.08 MPa, whereas the kirigami‐patterned patches show a reduced Young's modulus of 0.62 MPa and a peeling force of 2.12 MPa, corresponding to approximately 53 % and 35 % of the values measured for the plain design, respectively. The reduced modulus indicates enhanced ability to release tensile stress through structural deformation, while the moderated peeling force reflects improved conformability without excessive adhesion. A detailed comparison of Young's modulus and peeling force between plain and kirigami patches is depicted in Figure S5. These mechanical characteristics enable stable, conformal electrode‐skin contact under dynamic forehead motion. Overall, the theoretical analysis and mechanical characterization establish a wrinkle‐adaptive, strain‐decoupling mechanical framework that stabilizes the electrode‐skin interface, providing the structural foundation for the enhanced EEG signal quality demonstrated in the following section.

### 2.3 Enhanced EEG Signals Enabled by Personalized Kirigami Patches

**FIGURE 4:**
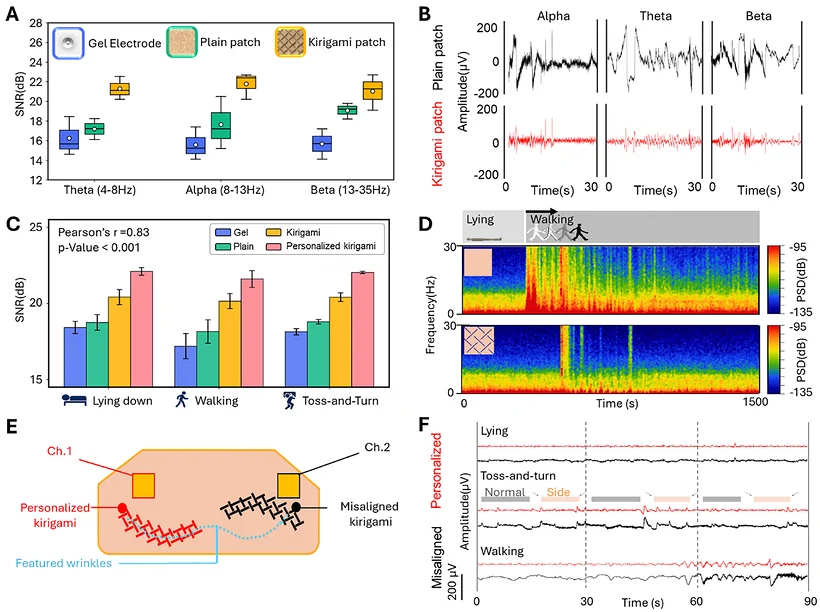
Architecture of an automated personalized kirigami patch during motion. (A) Signal‐to‐noise ratio (SNR) of three different electrodes across frequency bands, according to the criteria of the American Association of Sleep Medicine. (B) Comparison of raw EEG signal from both plain patch and kirigami patch on frequency ranges; signals were measured simultaneously from the same subject. (C) Signal‐to‐noise ratio of four different electrodes within three motions (Lying down, Walking, and Toss‐and‐turn). (D) Spectrogram of the plain and kirigami patch during lying down and then walking. (E) Schematic image showing the setup for simultaneously comparing personalized kirigami and misaligned kirigami. (F) Raw signal of each of the two types of patches monitored during lying, toss‐and‐turn, and walking for 90 s (Red: personalized kirigami, Black: misaligned kirigami).

The mechanically stabilized electrode‐skin interface enabled by the kirigami architecture directly translates into improved EEG signal quality under both static and dynamic conditions. Figure 4A compares the signal‐to‐noise ratio (SNR) obtained using gel electrodes, plain patches, and kirigami patches across the multiple EEG frequency bands. Details of the experimental setup appear in Figure S6. Kirigami patches consistently exhibit higher SNR values over the theta (4–7 Hz), alpha (8–13 Hz), and beta (14–30 Hz) bands compared to both gel electrodes and plain patches. Averaged across all frequency ranges, the SNR values are 15.83 ± 1.54 dB for gel electrodes, 17.96 ± 1.72 dB for plain patches, and 21.37 ± 1.32 dB for kirigami patches, demonstrating a clear enhancement in signal fidelity enabled by the kirigami structure. To provide additional quantitative support for interfacial stabilization, electrode–skin contact impedance characteristics were analyzed (Figure S7). Impedance measurements were performed within the primary frequency range of interest (1–100 Hz), comparing gold FPC electrodes integrated into plain medical patches with identical solid electrodes integrated into kirigami‐patterned patches. As shown in Figure S7A, kirigami‐patterned electrodes consistently exhibited lower electrode–skin contact impedance across the measured frequency spectrum, indicating more stable interfacial contact. Furthermore, impedance variability was quantified using the coefficient of variation derived from triplicated measurements. As summarized in Figure S7B, kirigami patches exhibited reduced impedance variability compared to plain patches, indicating improved mechanical stabilization at the electrode–skin interface. This reduced variability supports enhanced interfacial stability, which contributes to improved signal robustness. Representative raw EEG signals recorded simultaneously using plain and kirigami patches are shown in Figure 4B. Compared to plain patches, kirigami patches exhibit markedly reduced baseline drift and signal fluctuation across all frequency bands. Quantitatively, the peak‐to‐peak amplitude of raw EEG signals recorded using plain patches is 255 %, 163 %, and 234 % of that measured using kirigami patches in the theta, alpha, and beta bands, respectively. The absence of pronounced low‐frequency drift and high‐frequency fluctuations in kirigami patches is consistent with improved stability of the electrode–skin interface, whereas plain patches show intermittent signal instability indicative of motion‐induced contact variation. These comparisons highlight the effectiveness of the kirigami pattern in preserving signal integrity at the raw‐data level, prior to any post‐processing. Although kirigami structures substantially improve signal stability, the forehead presents additional challenges due to inter‐individual variations in skin roughness and wrinkle morphology. Facial motion and involuntary movements, particularly during sleep, can locally alter skin topography and induce gaps at the electrode‐skin interface. To address this variability, personalized kirigami structures were implemented by aligning kirigami patterns with individual wrinkle features, with the goal of further stabilizing electrode contact under realistic motion conditions. The performance of kirigami and personalized kirigami patches was evaluated under representative motion scenarios, including lying down, walking, and toss‐and‐turning movements, as summarized in Figure 4C. Across all electrode configurations, continuous walking resulted in a pronounced reduction in SNR relative to the lying condition, reflecting the impact of sustained body motion. For example, the average SNR during walking decreased to 14.37 ± 1.50 dB for gel electrodes, 16.29 ± 1.40 dB for plain patches, and 20.29 ± 0.90 dB for kirigami patches. In contrast, personalized kirigami patches maintained a higher SNR of 23.20 ± 1.00 dB during walking, exhibiting the smallest performance degradation among all electrode types. Underlying and toss‐and‐turn conditions, which more closely resemble typical sleep‐related movements, personalized kirigami patches exhibited consistently high, reproducible SNR values (24.19 ± 0.50 dB during lying and 24.06 ± 0.46 dB across toss‐and‐turn conditions). When averaged across all tested motion conditions, the mean SNR values were 16.05 ± 0.75 dB for gel electrodes, 17.35 ± 1.07 dB for plain patches, 20.72 ± 0.73 dB for kirigami patches, and 23.94 ± 0.53 dB for personalized kirigami patches. Notably, SNR values across different electrode configurations showed a strong positive correlation with the degree of mechanical conformity (r = 0.83, *p*< 0.001), indicating that improved mechanical adaptation to forehead motion directly translates into enhanced EEG signal quality. Time‐frequency analysis further demonstrates the motion robustness conferred by personalized kirigami designs. As shown in Video S1, the personalized kirigami patches closely conform to subject‐specific forehead wrinkle patterns, enabling conformal contact at the skin‐electrode interface. The fine‐scale kirigami features deform cooperatively with the skin, allowing local folding and strain accommodation during forehead wrinkling and motion. As a result, motion‐induced artifacts associated with skin deformation are mechanically attenuated at the interface, leading to improved signal stability and enabling continuous forehead EEG monitoring. Figure 4D presents spectrograms recorded during a transition from lying down to walking, using both a plain and a personalized kirigami patch. While both patches showed comparable spectral characteristics during the lying condition, the plain patch exhibits broadband spectral disturbances during walking, particularly above 10 Hz. In contrast, the personalized kirigami patch shows substantially suppressed motion‐related spectral components, consistent with reduced transmission of mechanical disturbance to the electrode‐skin interface. Finally, the effect of personalization was directly examined with a single patch configuration. Figure 4E shows raw EEG signals acquired simultaneously from two regions of the same patch: one region where the kirigami pattern is aligned with extracted wrinkle features, and another region with a kirigami pattern of identical feature size and geometry but misaligned relative to the wrinkles. The wrinkle‐aligned personalized kirigami region exhibits consistently lower noise levels and greater signal stability across lying, toss‐and‐turning, and walking conditions. This direct comparison confirms that, beyond kirigami patterning alone, aligning the kirigami architectures with individual wrinkle morphologies plays a critical role in stabilizing the electrode‐skin interface under dynamic conditions. The results in Figure 4F demonstrate that kirigami‐based patches substantially enhance EEG signal quality by mitigating motion‐induced artifacts, and that personalizing the kirigami architecture provides an additional level of robustness under realistic, demanding motion scenarios. These improvements in raw signal fidelity form the basis for reliable long‐term EEG monitoring, as further demonstrated in subsequent application studies.

### 2.4 Long‐Term At‐Home Sleep EEG Monitoring

**FIGURE 5:**
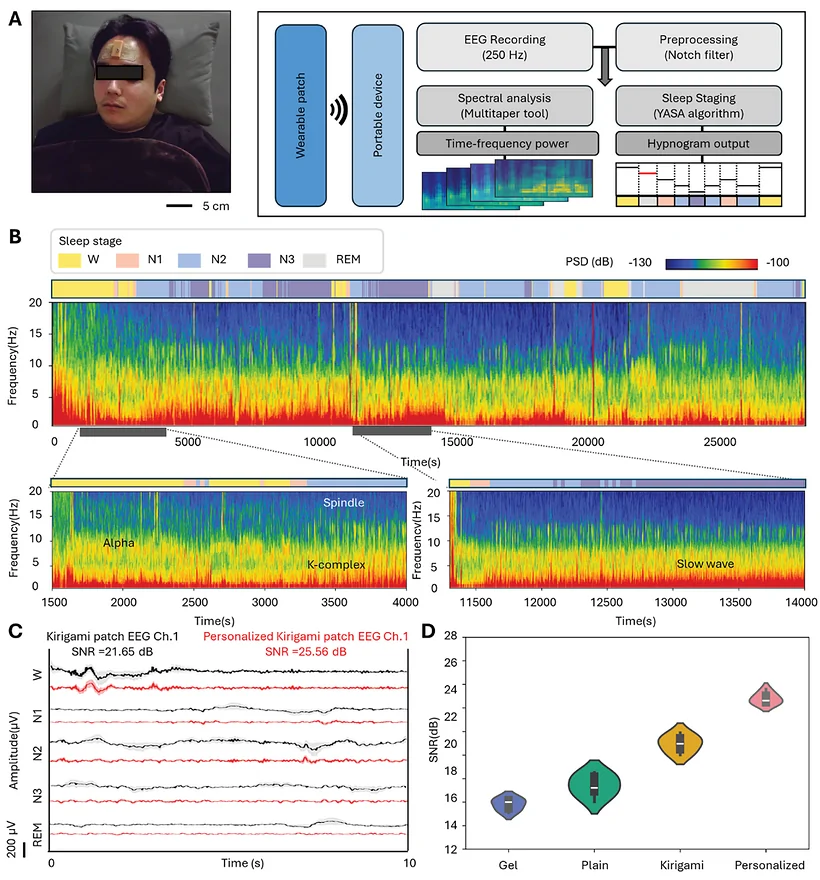
Measurement of sleep stages and comparison of signal quality. (A) Image of a healthy subject wearing a sleep patch and schematic illustration of the wearable EEG system. (B) Long‐term time–frequency spectrogram of an overnight EEG recording. Characteristic sleep‐related frequency patterns appear across the night, with corresponding hypnogram labels shown above the spectrogram. (C) Raw EEG signals of the kirigami patch and the personalized Kirigami patch, simultaneously. (D) SNR comparison across different electrode types (Gel, Plain, Kirigami, Personalized).

Building on the mechanically stabilized and motion‐robust electrode–skin interface, the performance of the personalized kirigami EEG patch was further evaluated during overnight sleep monitoring. Figure 5A shows an image of a subject wearing the personalized kirigami patch during sleep, together with a schematic of the signal acquisition and processing pipeline. The patch is conformally attached to the forehead, and EEG signals are transmitted wirelessly to a portable device for continuous recording. Acquired EEG signals were preprocessed using a notch filter and analyzed using multitaper‐based time‐frequency spectral analysis, followed by automated sleep staging with the YASA algorithm according to AASM criteria. Figure 5B presents the time‐frequency spectrogram and corresponding hypnogram obtained over approximately 8 h of sleep using the personalized kirigami patch (details in Figure S8). Distinct sleep‐stage–specific neural features are clearly resolved, including alpha activity during wakefulness and light sleep, K‐complexes and sleep spindles during N2 sleep, and prominent slow‐wave activity during N3 sleep. The sleep staging analysis was performed using the YASA algorithm. Its performance was independently validated using publicly available datasets with expert‐labeled sleep stages, yielding high agreement across all stages (Figure S9). To directly compare signal stability during sleep, raw EEG signals acquired simultaneously using a kirigami patch and a personalized kirigami patch were analyzed across different sleep stages (Figure 5C). The personalized kirigami patch consistently exhibits a higher signal‐to‐noise ratio (25.19 dB) than the non‐personalized kirigami patch (21.65 dB). Across all sleep stages except wakefulness, the personalized kirigami patch shows stable waveforms with peak‐to‐peak amplitudes within 44–80 µV, whereas the non‐personalized kirigami patch exhibits larger amplitude fluctuations and baseline instability, indicative of intermittent contact variation during sleep‐related motion. In addition to improved signal stability, characteristic sleep‐stage‐specific EEG features, including theta activity in N1, K‐complexes in N2, and slow‐wave activity in N3 sleep, were clearly identified in the raw EEG signals, as shown in Figure S10. A summary of SNR values obtained during sleep for different electrode configurations is shown in Figure 5D. While commercial gel electrodes and plain fabric‐based patches provide measurable EEG signals, kirigami‐patterned patches exhibit improved SNR and consistency. When averaged across sleep, the SNR values increase from 15.8 ± 0.49 dB for gel electrodes and 17.41 ± 0.91 dB for plain patches to 20.95 ± 0.70 dB for kirigami patches, with the highest SNR of 24.78 ± 0.47 dB achieved using personalized kirigami patches. These results demonstrate that aligning the kirigami architecture with individual forehead wrinkle morphology provides an additional performance advantage beyond kirigami patterning alone. A comparative summary of representative wearable EEG systems (Table S1) indicates that most prior approaches primarily mitigate motion artifacts through multi‐stage algorithmic processing. In contrast, the present work suppresses noise structurally at the electrode–skin interface via a personalized kirigami design, thereby reducing reliance on complex post‐processing. This distinction underscores the complementary trade‐offs between computationally driven artifact correction and mechanically stabilized signal acquisition.

## 3 Conclusion

In summary, we have developed a morphology‐aware, kirigami‐structured EEG patch that enables stable, high‐fidelity monitoring during both dynamic movements and long‐term use, especially during sleep. The two‐level kirigami architecture effectively decouples external strain from the electrode–skin interface while preserving intimate, conformal contact, thereby minimizing motion‐induced artifacts at the point of signal acquisition. Mechanical characterization and cyclic testing demonstrate reduced effective modulus, optimized adhesion, and robust durability of the kirigami substrate. Leveraging an automated image‐based design workflow, our study shows precise personalization of kirigami patterns to individual forehead wrinkle morphologies, further enhancing signal stability during motion. Integrated within a compact, wireless system, the personalized patch supports reliable, long‐term EEG acquisition and accurate identification of sleep‐stage‐specific neural features during at‐home sleep. Overall, this work establishes kirigami‐enabled, morphology‐adaptive structural design as a powerful strategy for advancing EEG‐detecting technology and provides a broadly applicable framework for the development of personalized, motion‐resilient bioelectronic interfaces.

## 4 Experimental Section

### 4.1 Materials and Apparatus

The wearable EEG patch was fabricated using biocompatible elastomeric and fabric‐based materials. Medical‐grade silicone adhesive (Silbione, Elkem) was used as the skin‐contacting adhesion layer, while an elastic nonwoven polyurethane medical fabric tape (9970T, 3 M) served as the compliant substrate. Gold dry electrodes were patterned on polyimide (PI) films and integrated with the kirigami‐structured adhesive–fabric composite. All kirigami patterns were introduced by laser perforation using a nano‐ and femtosecond laser machining system (PhotoLaser U4, LPKF, and Femtosecond laser micromachining system, Optec). Personalized kirigami designs were generated through an automated image‐based workflow. Forehead images were processed using ImageJ software to extract wrinkle features, followed by logical and probability‐based feature classification. The extracted features were converted into customized kirigami layouts using AutoCAD, enabling precise control of unit geometry and feature scaling prior to laser fabrication. Kirigami feature dimensions and surface morphology were characterized using a laser scanning microscope (VK‐X3050, Keyence, Japan). Mechanical properties of the patches, including peeling strength and Young's modulus, were measured using a universal testing machine (HZ‐1007E, MMS Technology, Republic of Korea). Static tensile and peeling tests were conducted at controlled displacement rates to quantify adhesion strength and effective modulus of the kirigami and non‐kirigami substrates. Cyclic deformation and repeatability tests were performed separately to evaluate robustness under repeated motion. A piezoelectric linear stage (M‐112.1DG, Physik Instrumente) driven by a DC motor controller (C‐863, Physik Instrumente) was used to apply controlled cyclic displacements that emulate forehead motion during facial expressions and sleep‐related movements. Motion profiles, including displacement amplitude and cycle number, were programmed and synchronized using LabVIEW software. Electrical characterization and signal‐related measurements were conducted using a precision source/measure unit (B2902A, Keysight Technologies). EEG data acquisition during on‐body experiments was performed using a portable wireless recording system (BioRadio, Great Lakes NeuroTechnologies), which enabled real‐time data transmission to external devices. All instruments were calibrated prior to the experiments according to the manufacturer's specifications.

### 4.2 Wrinkle Feature Extraction and Personalized Kirigami Design

The workflow for designing personalized kirigami structures is presented in Figure 2E. Individual forehead images are used as inputs to an automated design pipeline, through which wrinkle features are extracted and translated into subject‐specific kirigami layouts. The resulting designs are generated automatically and used directly for fabrication, enabling scalable production of personalized patches without manual intervention. Details regarding Image pre‐processing and wrinkle feature classification begin with acquiring images with a smartphone camera under ambient lighting conditions. Subsequently, feature extraction was performed using ImageJ. To match the effective membrane dimensions of the wearable patch, all images were resized to 300 × 200 pixels. Image pre‐processing involved systematic adjustment of brightness and threshold values to enhance wrinkle contrast while suppressing background texture. The resized image dimensions correspond to an effective spatial resolution of approximately 0.3–0.4 mm per pixel across the forehead region, which is sufficient to resolve wrinkle features with millimeter‐scale pitch while maintaining computational robustness. Brightness and threshold values were systematically varied within empirically determined ranges (brightness: ±20 % of the original intensity; threshold: Otsu‐based threshold ±15 %) to account for illumination variability and skin texture heterogeneity. For each image, duplicated image sets were generated by varying the threshold and brightness conditions. Each set consisted of 10 images within a defined brightness or threshold range, resulting in grouped image sets labeled as *Group‐t‐(1–10)* and *Group‐b‐(1–10)*. The pre‐processed images were converted into binary images to distinguish wrinkle‐related features in terms of white and black pixel distributions. Within each group, the degree of pixel overlap was quantified across the 10 images. Pixel overlap was defined as the ratio of consistently detected white pixels across the 10 images within each group, normalized to the total number of white pixels in the union set. Based on the overlap ratio of white pixels, the images were classified into three probability ranges: 0 %–30 %, 30 %–70 %, and 70 %–100 %, which were labeled as *Group‐(t or b)‐(1–10)‐p‐1*, *Group‐(t or b)‐(1–10)‐p‐2*, and *Group‐(t or b)‐(1–10)‐p‐3*, respectively. As a subsequent step after the image pre‐processing, logical processing and kirigami feature assignment have been conducted. To identify robust wrinkle structures, logical image operations were applied to the probability‐classified image groups. Binary images within the *p‐3* groups were processed using logical operations (AND, OR, and NOR) in ImageJ to extract consistently overlapping wrinkle features. AND operations were used to extract conservative, highly consistent wrinkle features, whereas OR and NOR operations were employed to capture complementary structures and suppress background artifacts. Pixels that were perfectly matched across the *p‐3* groups were defined as primary wrinkle features and labeled as *wrinkle structures‐1*. To further capture secondary wrinkle features with moderate confidence, the *p‐1* and *p‐2* groups were reprocessed into a second‐level probability grouping (*p‐p* groups). The *p‐p‐3* subgroup generated through this process was subjected to the same logical operations, and the resulting features were labeled as *wrinkle structures‐2*. The final wrinkle map was obtained by combining *wrinkle structures‐1* and *wrinkle structures‐2*. This composite wrinkle map was transferred onto a black‐and‐white grid‐embedded image and imported into AutoCAD software for subsequent kirigami layout generation. All image processing and logical operations were automated using ImageJ macros, enabling reproducible, scalable extraction of the wrinkle feature without manual intervention.

### 4.3 Fabrication of Kirigami‐Structured Fabric

To fabricate the kirigami‐structured adhesive–fabric substrate, a soft silicone adhesive layer was first prepared on a poly(tetrafluoroethylene) (PTFE) carrier [3, 53, 54, 55]. Parts A and B of the silicone adhesive (Silbione, A‐4717, Factor II Inc.) were mixed at a 1:1 weight ratio for 10 min to ensure homogeneity. The mixture was poured onto a PTFE sheet and spin‐coated at 550 rpm for 60 s to form a uniform adhesive layer. An elastic nonwoven polyurethane medical fabric tape (9970T, 3 M) was laminated onto the uncured adhesive layer and subsequently cured in an oven at 65°C for 30 min. After curing, the PTFE carrier was peeled off to obtain a free‐standing adhesive–fabric composite substrate. Kirigami patterns were designed using AutoCAD software and introduced into the adhesive–fabric composite using nanosecond laser micromachining (PhotoLaser U4, LPKF, and Optec). Laser cutting was performed through both the adhesive layer and the fabric substrate to generate diagonal kirigami patterns with well‐defined slit geometry.

### 4.4 Device Assembly and Encapsulation

Gold dry electrodes patterned on polyimide (PI) films were laminated onto the kirigami‐structured adhesive–fabric substrate at predefined electrode locations. Electrical interconnections were formed between the electrodes and a flexible printed circuit board (fPCB) using conductive adhesives. The assembled device was fully encapsulated using a low‐modulus silicone elastomer (Ecoflex 00–30, Smooth‐On). Encapsulation was performed by mold casting using a custom‐designed mold fabricated by stereolithography (SLA) 3D printing (Form 3+, Formlabs). The encapsulated devices were cured in an oven at 65°C for 30 min to form a mechanically robust and electrically insulated forehead EEG patch suitable for on‐skin operation.

### 4.5 Mechanical Characterization and Cyclic Deformation Tests

Static mechanical properties of the patches, including Young's modulus and peeling strength, were measured using a universal testing machine (HZ‐1007E, MMS Technology, Republic of Korea). Tensile tests were performed at a constant displacement rate, and stress–strain curves were obtained to extract the effective Young's modulus. Peeling tests were conducted to quantify interfacial adhesion between the patch and a compliant substrate. Cyclic deformation and repeatability tests were performed separately to evaluate durability under repeated motion. A piezoelectric linear stage (M‐112.1DG, Physik Instrumente) driven by a DC motor controller (C‐863, Physik Instrumente) was used to apply cyclic tensile deformation. The samples were stretched to 20 % strain and released for up to 2000 cycles at a speed of 120 mm/min. Motion profiles and cycle control were programmed in LabVIEW.

### 4.6 EEG Acquisition and Signal Analysis

EEG signals were recorded from the forehead using the kirigami‐based forehead EEG patch and a commercial gel‐electrode system (BioRadio, Great Lakes NeuroTechnologies) as a reference. Electrodes were positioned over the frontal pole region, approximately corresponding to the Fpz location in the international 10–20 system, with reference and ground electrodes placed near Fp1 and Fp2. Signals were sampled at 250 Hz and wirelessly transmitted to a portable device. Raw EEG signals were preprocessed using a 60 Hz notch filter to remove power‐line interference. Time–frequency representations were obtained using a multitaper spectral analysis method. Sleep staging was performed using the YASA algorithm following AASM criteria [3, 56]. Signal quality was quantified using amplitude‐based signal‐to‐noise ratio (SNR) under different motion and sleep conditions. For band‐specific SNR evaluation, EEG signals were acquired under controlled behavioral conditions designed to elicit dominant activity in canonical frequency bands, including theta, alpha, and beta. For each band, a corresponding baseline recording was acquired to estimate noise under a matched posture and viewing condition. Theta band (4–7 Hz). Participants lay supine on a mattress with eyes closed for 5 min to elicit theta‐dominant resting activity. Recordings were initiated after a 30 min adaptation period to minimize transient effects. Noise for the theta‐band condition was estimated from a baseline recording acquired in the same posture with eyes open for 5 min immediately prior to the eyes‐closed recording. Alpha band (8–13 Hz). Participants sat on a chair with eyes closed for 5 min to elicit alpha activity. Noise for the alpha‐band condition was estimated from a baseline recording acquired in the same seated posture with eyes open for 5 min. Beta band (14–30 Hz). Beta activity was elicited using a cognitive stress task (Stroop test) to induce sustained mental engagement. Noise for the beta‐band condition was estimated from a baseline condition in which participants maintained their eyes open for 5 min while gazing at a uniform green background on a computer screen. To assess the robustness of the wearable forehead EEG system in practical use environments, SNR was further evaluated under representative motion conditions. To evaluate robustness under real‐world motion, SNR was additionally measured under three representative motion scenarios: lying down, walking, and toss‐and‐turning. For each scenario, recordings were acquired using the same acquisition settings and the same SNR definition described below.

- Lying down: Participant lying supine with eyes closed (stable baseline condition).
- Walking: Participant walking continuously (sustained whole‐body motion).
- Toss‐and‐turning: Participant performing sudden posture changes while lying down to emulate sleep‐related movements.

The aforementioned signal‐to‐noise ratio (SNR) for each condition has been defined in this research and then calculated on an amplitude basis for each EEG frequency band. For a given frequency band, SNR was defined as:

(1) $$SNR\left(dB\right)=20\cdot log10\left(\frac{A_{Signal}}{A_{Noise}}\right)$$

where *A*_signal_is the mean peak‐to‐peak amplitude obtained under the activation condition for the target band, and *A*_noise_is the mean peak‐to‐peak amplitude obtained under the corresponding baseline condition. This procedure was applied consistently across electrode configurations (gel electrodes, plain patches, kirigami patches, and personalized kirigami patches).

### 4.7 Sleep Staging and Data Processing

Overnight sleep EEG recordings were conducted using the personalized kirigami patches. Acquired signals were preprocessed using a notch filter (60 Hz) to remove power‐line interference and subjected to multitaper‐based time–frequency analysis [3, 56, 57, 58]. Automated sleep staging was performed using the YASA algorithm in accordance with the American Academy of Sleep Medicine (AASM) criteria. To validate the reliability of the sleep staging pipeline, the algorithm was evaluated using publicly available datasets with expert‐labeled sleep stages, and the corresponding confusion matrix is provided in the Supporting Information.

### 4.8 Biocompatibility Evaluation

Standard cell culture equipment, including a CO_2_ incubator with humidity control, water bath, centrifuge, and sterile work cabinet, was utilized. Cell viability was assessed using MTS assays with absorbance measurements taken on a 96‐well plate reader (Bio‐Teck, USA). L929 cells were cultured in EMEM supplemented with 10 % FBS and 1 % PS at 37°C. For cytotoxicity evaluation of the electrode patches, the electrode samples were soaked in culture medium for 24 h to create the test extract medium. L929 cells were seeded at a density of 1.0 × 10^5^ cells/mL in 96‐well plates and allowed to attach on surface overnight. The growth medium was then replaced with either the electrode‐extracted medium or the control medium. The experimental setup included positive controls (complete medium with 1 % SDS) and negative controls [59, 60]. Following a 72 h exposure period, MTS reagent (20 µL per well) was added and allowed to react for 1 h before measuring optical density at 490 nm. All experiments were performed with four replicates, and the data were standardized against the negative control values.

### 4.9 Human Subject Study

Three healthy male volunteers (n = 3; mean age 28.7 ± 2.5 years) participated in the study. The participants had no history of diseases that could affect the experimental results. The experimental protocol (#H20211) was approved by the Georgia Tech Institutional Review Board, ensuring compliance with ethical research standards. In accordance with ethical guidelines, all participants provided written informed consent prior to the study. They were given a detailed explanation of the experimental protocol, potential risks, and their right to withdraw at any time.

## Author Contributions

Jungmin Kim, Ka Ram Kim, Seong J. Cho, and Woon‐Hong Yeo conceptualized the work. Jungmin Kim, Ka Ram Kim, Byeongjun Lee, and Cheoljeong Park performed the experiments. Jungmin Kim, Ka Ram Kim, and Cheoljeong Park analyzed the data. Jungmin Kim, Ka Ram Kim, Seong J. Cho, and Woon‐Hong Yeo wrote the manuscript.

## Conflicts of Interest

Georgia Tech has a pending patent application related to this work.

## Supporting information




**Supporting File 1**: advs74871‐sup‐0001‐SuppMat.pdf.

**Supporting File 2**: advs74871‐sup‐0002‐VideoS1.docx.

## Data Availability

The data that support the findings of this study are available from the corresponding author upon reasonable request.

## References

[1] T. N. Alotaiby , S. A. Alshebeili , T. Alshawi , I. Ahmad , and F. E. Abd El‐Samie , “EEG Seizure Detection And Prediction Algorithms: A Survey,” EURASIP Journal on Advances in Signal Processing 2014, no. 1 (2014): 183, 10.1186/1687-6180-2014-183.

[2] R. B. Damalerio , R. Lim , Y. Gao , T.‐T. Zhang , and M.‐Y. Cheng , “Development of Low‐Contact‐Impedance Dry Electrodes for Electroencephalogram Signal Acquisition,” Sensors 23, no. 9 (2023): 4453, 10.3390/s23094453. PMC10181682 37177657

[3] S. Kwon , H. S. Kim , K. Kwon , et al., Science Advances 9, no. 21 (2023): adg9671, 10.1126/sciadv.adg9671. PMC10208583 37224243

[4] L. L. Popa , H. Dragos , C. Pantelemon , O. V. Rosu , and S. Strilciuc , “The Role of Quantitative EEG in the Diagnosis of Neuropsychiatric Disorders,” Journal of medicine and life 13, no. 1 (2020): 8–15, 10.25122/jml-2019-0085. PMC7175442 32341694

[5] A. Défossez , C. Caucheteux , J. Rapin , O. Kabeli , and J.‐R. King , “Decoding Speech Perception From Non‐Invasive Brain Recordings,” Nature Machine Intelligence 5, no. 10 (2023): 1097–1107, 10.1038/s42256-023-00714-5.

[6] D. Zhang , Z. Wang , Y. Qian , et al., “A brain‐to‐text framework for decoding natural tonal sentences,” Cell Reports 43, no. 11 (2024): 114924, 10.1016/j.celrep.2024.114924. 39485790

[7] S. Saha , K. A. Mamun , K. Ahmed , et al., “Progress in Brain Computer Interface: Challenges and Opportunities,” Frontiers in Systems Neuroscience 15 (2021): 578875, 10.3389/fnsys.2021.578875. PMC7947348 33716680

[8] F. Costa , E. V. Schaft , G. Huiskamp , et al., “Robust Compression And Detection Of Epileptiform Patterns in ECoG Using A Real‐Time Spiking Neural Network Hardware Framework,” Nature Communications 15, no. 1 (2024): 3255, 10.1038/s41467-024-47495-y. PMC11021517 38627406

[9] A. Saibene , M. Caglioni , S. Corchs , and F. Gasparini , “EEG‐Based BCIs on Motor Imagery Paradigm Using Wearable Technologies: A Systematic Review,” Sensors 23, no. 5 (2023): 2798, 10.3390/s23052798. PMC10007053 36905004

[10] H. Kim , J. H. Kim , Y. J. Lee , et al., “Motion Artifact–Controlled Micro–Brain Sensors Between Hair Follicles For Persistent Augmented Reality Brain–Computer Interfaces,” Proceedings of the National Academy of Sciences 122, no. 15 (2025): 2419304122, 10.1073/pnas.2419304122. PMC12012477 40193612

[11] Z.‐Z. Ma , J.‐J. Wu , Z. Cao , et al., “Motor Imagery‐Based Brain–Computer Interface Rehabilitation Programs Enhance Upper Extremity Performance And Cortical Activation In Stroke Patients,” Journal of neuroengineering and rehabilitation 21, no. 1 (2024): 91, 10.1186/s12984-024-01387-w. PMC11134735 38812014

[12] M. Mahmood , S. Kwon , H. Kim , et al., “Wireless Soft Scalp Electronics and Virtual Reality System for Motor Imagery‐Based Brain–Machine Interfaces,” Advanced Science 8, no. 19 (2021): 2101129, 10.1002/advs.202101129. PMC8498913 34272934

[13] N. Avital , N. Shulkin , and D. Malka , “Automatic Calculation of Average Power in Electroencephalography Signals for Enhanced Detection of Brain Activity and Behavioral Patterns,” Biosensors 15, no. 5 (2025): 314, 10.3390/bios15050314. PMC12110619 40422053

[14] J.‐H. Kim , H. Nam , D. Won , and C.‐H. Im , “Domain‐generalized Deep Learning for Improved Subject‐independent Emotion Recognition Based on Electroencephalography,” Experimental Neurobiology 34, 3 (2025): 119–130, 10.5607/en25011. PMC12235040 40364497

[15] A. Hekmatmanesh , P. H. Nardelli , and H. Handroos , “Review of the State‐of‐the‐Art of Brain‐Controlled Vehicles,” IEEE Access 9 (2021): 110173–110193, 10.1109/ACCESS.2021.3100700.

[16] M. Mahmood , N. Kim , M. Mahmood , et al., “VR‐enabled portable brain‐computer interfaces via wireless soft bioelectronics,” Biosensors and Bioelectronics 210 (2022): 114333, 10.1016/j.bios.2022.114333. 35525171

[17] W. J. Bosl , H. Tager‐Flusberg , and C. A. Nelson , “EEG Analytics for Early Detection of Autism Spectrum Disorder: A data‐driven approach,” Scientific reports 8, no. 1 (2018): 6828, 10.1038/s41598-018-24318-x. PMC5931530 29717196

[18] J. S. Kim , E.‐S. Kang , Y. C. Bahk , S. Jang , K. S. Hong , and J. H. Baek , “Exploratory Analysis of Behavioral Impulsivity, Pro‐inflammatory Cytokines, and Resting‐State Frontal EEG Activity Associated With Non‐suicidal Self‐Injury in Patients With Mood Disorder,” Frontiers in psychiatry 11 (2020): 124, 10.3389/fpsyt.2020.00124. PMC7057238 32174860

[19] H. Kim , H. Kim , Y. J. Lee , et al., “Continuous Real‐Time Detection And Management Of Comprehensive Mental States Using Wireless Soft Multifunctional Bioelectronics,” Biosensors and Bioelectronics 279 (2025): 117387, 10.1016/j.bios.2025.117387. 40120293

[20] M. R. Carneiro , A. T. de Almeida , and M. Tavakoli , “Wearable and Comfortable E‐Textile Headband for Long‐Term Acquisition of Forehead EEG Signals,” IEEE Sensors Journal 20, no. 24 (2020): 15107–15116, 10.1109/JSEN.2020.3009629.

[21] A. D. Nordin , W. D. Hairston , and D. P. Ferris , “Human Electrocortical Dynamics While Stepping Over Obstacles,” Scientific reports 9, no. 1 (2019): 4693, 10.1038/s41598-019-41131-2. PMC6423113 30886202

[22] A. Giangrande , A. Botter , H. Piitulainen , and G. L. Cerone , “Motion Artifacts in Dynamic EEG Recordings: Experimental Observations, Electrical Modelling, and Design Considerations,” Sensors 24, no. 19 (2024): 6363, 10.3390/s24196363. PMC11479364 39409399

[23] Z. Liu , X. Xu , S. Huang , et al., “Multichannel Microneedle Dry Electrode Patches For Minimally Invasive Transdermal Recording Of Electrophysiological Signals,” Microsystems & Nanoengineering 10, no. 1 (2024): 72, 10.1038/s41378-024-00702-8. PMC11143369 38828404

[24] J. Li , Y. Ma , D. Huang , et al., “High‐Performance Flexible Microneedle Array as a Low‐Impedance Surface Biopotential Dry Electrode for Wearable Electrophysiological Recording and Polysomnography,” Nano‐Micro Letters 14, no. 1 (2022): 132, 10.1007/s40820-022-00870-0. PMC9198145 35699782

[25] L. Chen , H. Zhong , L. Wang , et al., “Long‐Term Stable Subdural Recordings Enabled by Fibrosis‐Resistant Hydrogel‐Integrated µECoG Arrays,” Advanced Science 12 (2025): 15453, 10.1002/advs.202515453. PMC12713035 41059974

[26] B. Ji , F. Sun , J. Guo , et al., “Brainmask: An Ultrasoft And Moist Micro‐Electrocorticography Electrode For Accurate Positioning And Long‐Lasting Recordings,” Microsystems & Nanoengineering 9, no. 1 (2023): 126, 10.1038/s41378-023-00597-x. PMC10564857 37829160

[27] C. Dong , A. Carnicer‐Lombarte , F. Bonafè , et al., “Electrochemically Actuated Microelectrodes For Minimally Invasive Peripheral Nerve Interfaces,” Nature Materials 23, no. 7 (2024): 969–976, 10.1038/s41563-024-01886-0. PMC11230894 38671159

[28] W. D. Hairston , K. W. Whitaker , A. J. Ries , et al., “Usability of Four Commercially‐Oriented EEG Systems,” Journal of neural engineering 11, no. 4 (2014): 046018, 10.1088/1741-2560/11/4/046018. 24980915

[29] M. Mehrali , S. Bagherifard , M. Akbari , et al., “Blending Electronics with the Human Body: A Pathway toward a Cybernetic Future,” Advanced Science 5, no. 10 (2018): 1700931, 10.1002/advs.201700931. PMC6193179 30356969

[30] T. Kawana , Y. Yoshida , Y. Kudo , C. Iwatani , and N. Miki , “Design and Characterization of an EEG‐Hat for Reliable EEG Measurements,” Micromachines 11, no. 7 (2020): 635, 10.3390/mi11070635. PMC7407528 32605330

[31] D. Seok , S. Lee , M. Kim , J. Cho , and C. Kim , “Motion Artifact Removal Techniques for Wearable EEG and PPG Sensor Systems,” Frontiers in Electronics 2 (2021): 685513, 10.3389/felec.2021.685513.

[32] P. J. Arnal , V. Thorey , E. Debellemaniere , et al., “The Dreem Headband Compared To Polysomnography For Electroencephalographic Signal Acquisition And Sleep Staging,” Sleep 43, no. 11 (2020): zsaa097, 10.1093/sleep/zsaa097. PMC7751170 32433768

[33] A. J. Casson , “Wearable EEG and Beyond,” Biomedical engineering letters 9, no. 1 (2019): 53–71, 10.1007/s13534-018-00093-6. PMC6431319 30956880

[34] L. Molcho , N. B. Maimon , N. Hezi , T. Zeimer , N. Intrator , and T. Gurevich , “Evaluation of Parkinson's Disease Early Diagnosis Using Single‐Channel EEG Features And Auditory Cognitive Assessment,” Frontiers in Neurology 14 (2023): 1273458, 10.3389/fneur.2023.1273458. PMC10762798 38174098

[35] E. López‐Larraz , C. Escolano , A. Robledo‐Menéndez , L. Morlas , A. Alda , and J. Minguez , “A Garment That Measures Brain Activity: Proof of Concept of an EEG Sensor Layer Fully Implemented With Smart Textiles,” Frontiers in Human Neuroscience 17 (2023): 1135153, 10.3389/fnhum.2023.1135153. PMC10250743 37305362

[36] H. Kim , H. Kim , S. Kang , et al., “Face‐Wearable Integrated Bioelectronics For Quantitative, Automated Diagnosis Of Blepharospasm,” Biosensors and Bioelectronics: X 26 (2025), 10.1016/j.biosx.2025.100677.

[37] Z. Gao , X. Cui , W. Wan , Z. Qin , and Z. Gu , “Signal Quality Investigation of a New Wearable Frontal Lobe EEG Device,” Sensors 22, no. 5 (2022): 1898, 10.3390/s22051898. PMC8914983 35271044

[38] N. L. Wickramasinghe , D. S. Udayantha , A. Abeyratne , et al., “An Active Dry‐Contact Continuous EEG Monitoring System for Seizure Detection Applications in Clinical Neurophysiology,” IEEE Transactions on Biomedical Engineering (2025): 1–10, 10.1109/TBME.2025.3629563. 41196782

[39] A. Bertrand , V. Mihajlović , B. Grundlehner , C. Van Hoof , and M. Moonen , “Motion Artifact Reduction in EEG Recordings Using Multi‐Channel Contact Impedance Measurements,” in 2013 IEEE Biomedical Circuits and Systems Conference (BioCAS) (IEEE, 2013), 258–261, 10.1109/BioCAS.2013.6679688.

[40] K. Zheng , C. Zheng , L. Zhu , et al., “Machine Learning Enabled Reusable Adhesion, Entangled Network‐Based Hydrogel for Long‐Term, High‐Fidelity EEG Recording and Attention Assessment,” Nano‐Micro Letters 17, no. 1 (2025): 281, 10.1007/s40820-025-01780-7. PMC12122957 40439842

[41] H. Li , H. Niu , F. Yin , W. Zhou , G. Shen , and Y. Li , “A Labor‐Division Cooperation Electronic Palm System for High‐Precision Crosstalk‐Free Cognition of Pressure and Temperature,” Advanced Materials 38, no. 8 (2026): 10241, 10.1002/adma.202510241. 41307294

[42] Y. Li , Q. Lin , T. Sun , M. Qin , W. Yue , and S. Gao , “A Perceptual and Interactive Integration Strategy Toward Telemedicine Healthcare Based on Electroluminescent Display and Triboelectric Sensing 3D Stacked Device,” Advanced Functional Materials 34, no. 40 (2024), 10.1002/adfm.202402356.

[43] Y. Li , Z. Qiu , H. Kan , et al., “A Human‐Computer Interaction Strategy for An FPGA Platform Boosted Integrated “Perception‐Memory” System Based on Electronic Tattoos and Memristors,” Advanced Science 11, no. 39 (2024): 2402582, 10.1002/advs.202402582. PMC11497050 39049180

[44] B. Shin , Y. Kwon , M. Mittaz , et al., “All‐In‐One Wearable Drug Efficacy Assessment Systems For Bulbar Muscle Function Using Amyotrophic Lateral Sclerosis Animal Models,” Nature Communications 15, no. 1 (2024), 10.1038/s41467-024-51300-1. PMC11315987 39122743

[45] B. Shin , S. H. Lee , K. Kwon , et al., “All‐In‐One Wearable Drug Efficacy Assessment Systems For Bulbar Muscle Function Using Amyotrophic Lateral Sclerosis Animal Models,” Advanced Science 11, no. 34 (2024): 2404211, 10.1002/advs.202404211. PMC11315987 39122743

[46] X. Qian , M. Wang , X. Wang , Y. Wang , and W. Dai , “Intelligent Method for Real‐Time Portable EEG Artifact Annotation in Semiconstrained Environment Based on Computer Vision,” Computational Intelligence and Neuroscience 2022, no. 1 (2022):9590411, 10.1155/2022/9590411. PMC8858064 35190736

[47] V. Bono , S. Das , W. Jamal , and K. Maharatna , “Hybrid Wavelet and EMD/ICA Approach for Artifact Suppression in Pervasive EEG,” Journal of Neuroscience Methods 267 (2016): 89–107, 10.1016/j.jneumeth.2016.04.006. 27102040

[48] M. Rusanen , S. Myllymaa , L. Kalevo , et al., “An In‐Laboratory Comparison of FocusBand EEG Device and Textile Electrodes Against a Medical‐Grade System and Wet Gel Electrodes,” IEEE Access 9 (2021): 132580–132591, 10.1109/ACCESS.2021.3113049.

[49] T. Liu , X. Hu , D. Wang , et al., “Flexible dry electrodes for electrocardiographic monitoring: Working principles, design strategies, and recent advances,” Iscience 28 11 (2025): 113812, 10.1016/j.isci.2025.113812. PMC12616126 41244578

[50] A. Veronica , H. Y. Y. Nyein , and I.‐M. Hsing , “Ionogels and Eutectogels for Stable and Long‐Term EEG and EMG Signal Acquisition,” Materials Futures 3, no. 3 (2024): 033501, 10.1088/2752-5724/ad5c84.

[51] A. B. M. T. Haque , D. Hwang , and M. D. Bartlett , “Graded Kirigami Composites for Programmed Strain Distributions,” Advanced Materials Technologies 7, no. 7 (2022): 2101241, 10.1002/admt.202101241.

[52] B. Shin , Y. Kwon , M. Mittaz , et al., “All‐In‐One Wearable Drug Efficacy Assessment Systems For Bulbar Muscle Function Using Amyotrophic Lateral Sclerosis Animal Models,” Nature Communications 15, no. 1 (2024): 6803, 10.1038/s41467-024-51300-1. PMC11315987 39122743

[53] H. Kim , H.‐S. Cha , M. Kim , et al., “AR‐Enabled Persistent Human–Machine Interfaces via a Scalable Soft Electrode Array,” Advanced Science 11 (2024): 2305871, 10.1002/advs.202305871. PMC10870043 38087936

[54] J. Kim , P. Kantharaju , H. Yi , et al., “Soft Wearable Flexible Bioelectronics Integrated With An Ankle‐Foot Exoskeleton For Estimation Of Metabolic Costs And Physical Effort,” npj Flexible Electronics 7, no. 1 (2023): 3, 10.1038/s41528-023-00239-2.

[55] J. Lee , K. Kwon , I. Soltis , et al., “Intelligent Upper‐Limb Exoskeleton Integrated With Soft Bioelectronics And Deep Learning For Intention‐Driven Augmentation,” npj Flexible Electronics 8, no. 1 (2024): 11, 10.1038/s41528-024-00297-0.

[56] S. Ban , Y. Kwon , I. Shin , et al., “Breathable Soft Bioelectronics For Enhanced Automatic Detection Of Obstructive Sleep Apnea,” Biosensors and Bioelectronics 287 (2025): 117735, 10.1016/j.bios.2025.117735. 40609200

[57] S. Kwon , H. Kim , and W.‐H. Yeo , “Recent Advances In Wearable Sensors And Portable Electronics For Sleep Monitoring,” iScience 24, no. 5 (2021): 102461, 10.1016/j.isci.2021.102461. PMC8113882 34013173

[58] K. Kwon , S. Kwon , and W.‐H. Yeo , “Automatic and Accurate Sleep Stage Classification via a Convolutional Deep Neural Network and Nanomembrane Electrodes,” Biosensors 12, no. 3 (2022): 155, 10.3390/bios12030155. PMC8946692 35323425

[59] A. Bateman , G. Byun , S. Oh , et al., “Wireless, Battery‐Free Self‐Detecting Smart Arteriovenous Graft For Stenosis Diagnosis In Dialysis Patients,” Biosensors and Bioelectronics 293 (2026): 118088, 10.1016/j.bios.2025.118088. 41135237

[60] C. Ogbonna , Y. Kwon , K. R. Kim , W.‐H. Yeo , N. Ghalichechian , and L. Beardslee , “A Biomaterial‐Based Approach to Trypsin Sensing: Design and Optimization of Gelatin–Casein Films,” ACS Omega 10 (August, 2025): 37597–37610, 10.1021/acsomega.5c03938. PMC12391974 40893331

