# Supplementary material for: Wrinkle‐Adaptive Kirigami Wearables With Anisotropic Deformability for Sleep EEG Monitoring
Source: Adv Sci (Weinh). 2026 Mar 20;13(31):e00035. doi: 10.1002/advs.202600035 (PMC13587928; doi:10.1002/advs.202600035)
Supplement: Supplementary file 1 [file ADVS-13-e00035-s001.pdf]

# Supporting Information

**Wrinkle-Adaptive Kirigami Wearables with Anisotropic Deformability for Sleep EEG Monitoring**

*Jungmin Kim†, Ka Ram Kim†, Byeongjun Lee, Cheoljeong Park, Seong J. Cho*, Woon-Hong Yeo**

Jungmin Kim†, Byeongjun Lee, Cheoljeong Park, Seong J. Cho*
Department of Mechanical Engineering, Chungnam National University, Daejeon, 34134, Republic of Korea
E-mail: scho@cnu.ac.kr

Ka Ram Kim†
Wearable Intelligent Systems and Healthcare Center (WISH Center) at the Institute for Matter and Systems, Georgia Institute of Technology, Atlanta, GA 30332, USA
Korea KIAT-Georgia Tech Semiconductor Electronics Center at the Institute for Matter and Systems, Georgia Institute of Technology, Atlanta, GA 30332, USA

Woon-Hong Yeo*
George W. Woodruff School of Mechanical Engineering, Georgia Institute of Technology, Atlanta, GA 30332, USA
Wearable Intelligent Systems and Healthcare Center (WISH Center) at the Institute for Matter and Systems, Georgia Institute of Technology, Atlanta, GA 30332, USA
Wallace H. Coulter Department of Biomedical Engineering, Georgia Institute of Technology and Emory University School of Medicine, Atlanta, GA 30332, USA
Parker H. Petit Institute for Bioengineering and Biosciences, Georgia Institute of Technology, Atlanta, GA 30332, USA
Korea KIAT-Georgia Tech Semiconductor Electronics Center at the Institute for Matter and Systems, Georgia Institute of Technology, Atlanta, GA 30332, USA
E-mail: whyeo@gatech.edu

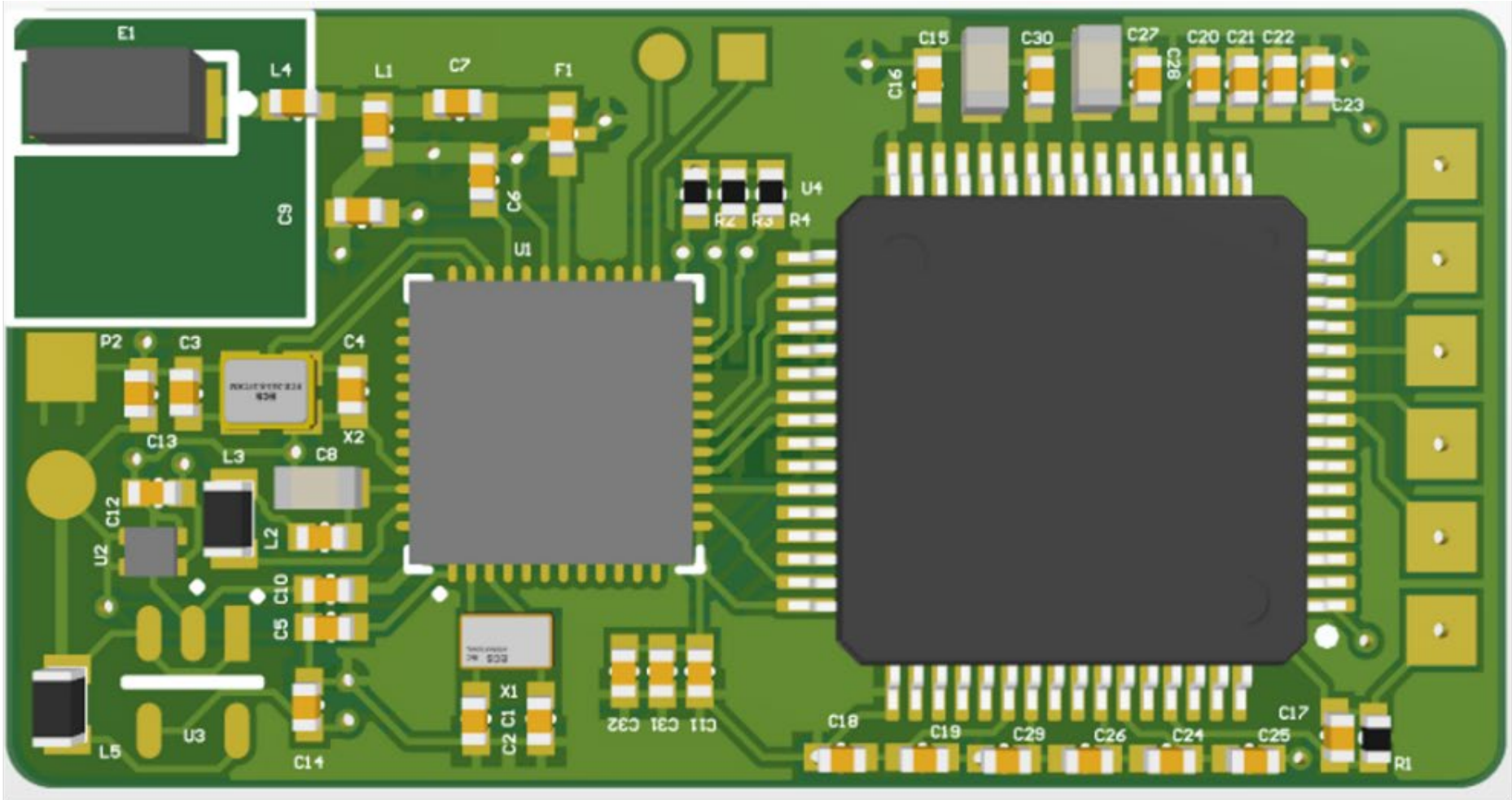

| Component | Manufacturer | Purpose | Quantity |
|---|---|---|---|
| ADS1299 | Analog Devices | Multiplexer | 1 |
| NRF52832 | Nordic Semiconductor | Microcontroller | 1 |
| TPS62746 | Texas Instruments | Voltage regulator | 1 |
| TPS61222 | Texas Instruments | Voltage regulator | 1 |

**Figure S1.** Summary of IC components for the EEG patch.

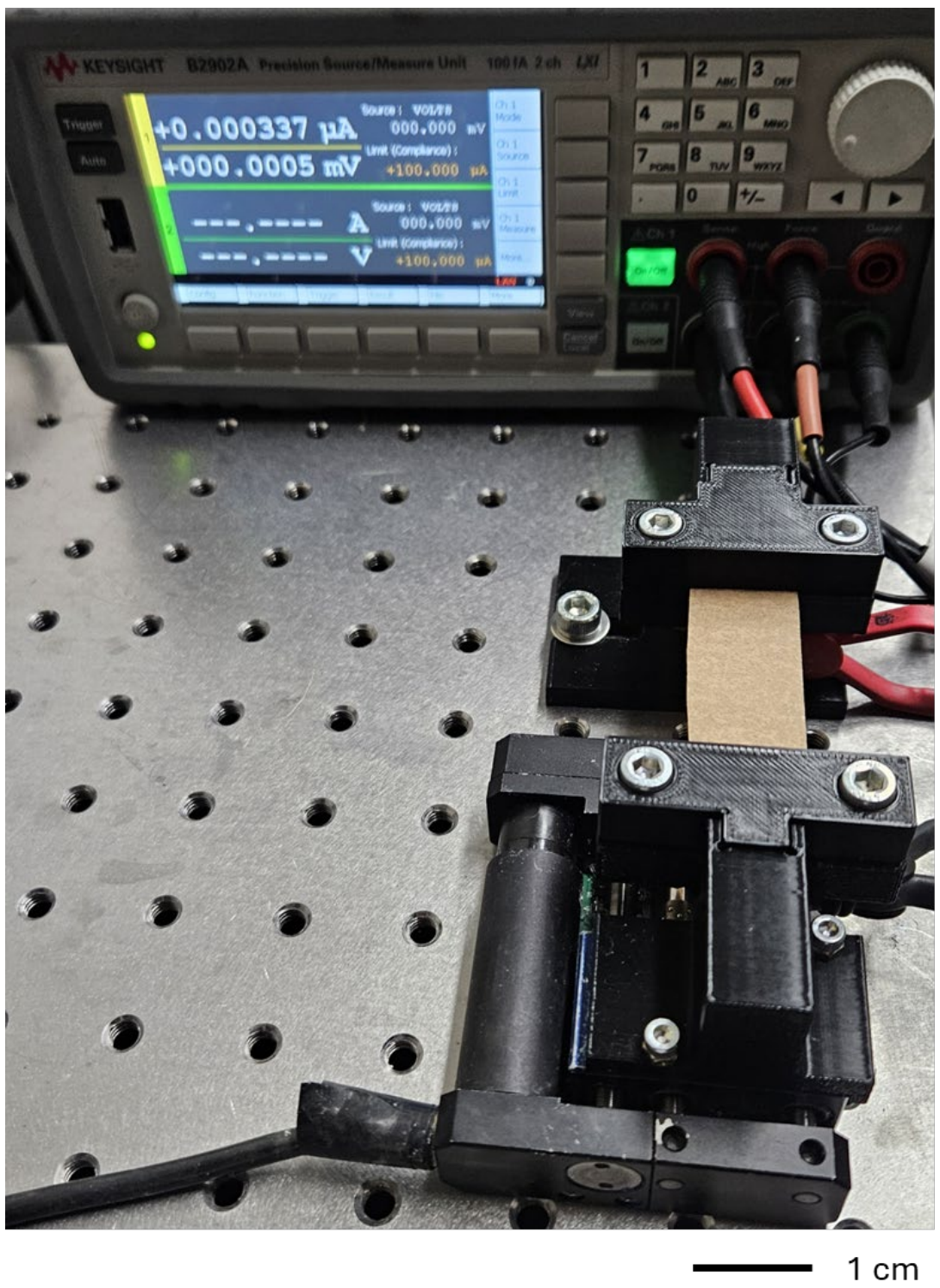


**Figure S2.** Experimental setup for resistance monitoring of integrated electrodes with a patch throughout the cyclic stretching test.

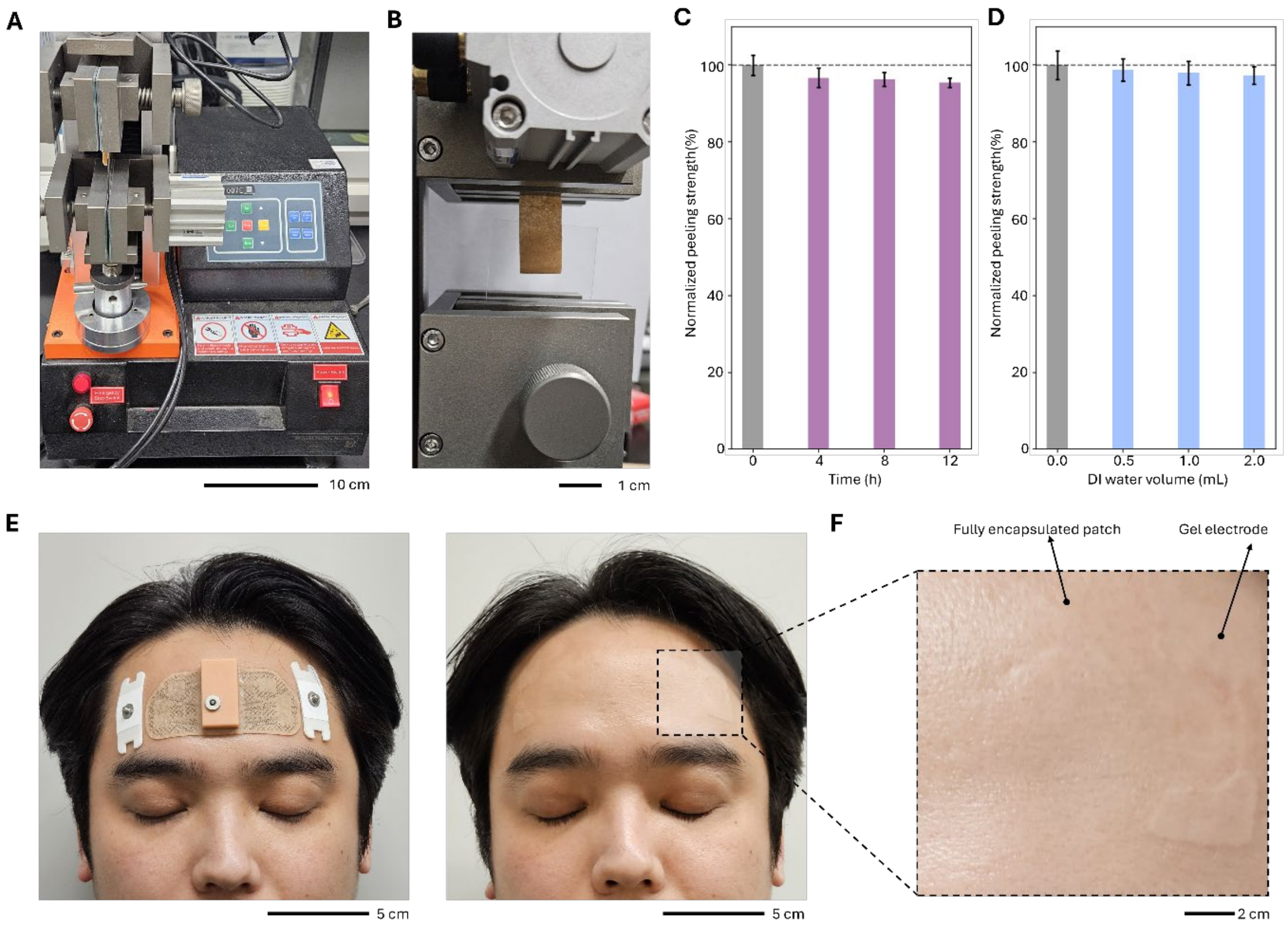


**Figure S3**. Interfacial adhesion stability and long-term wear validation of the fully encapsulated personalized kirigami patch. (A) Experimental setup for 180° peeling tests using a Universal Testing Machine. (Model 1007E, MMS) (B) Optical image of patches being peeled off from the PDMS substrate coated on glass. (C) Normalized peeling strength of the kirigami patch from skin as a function of attachment duration (0, 4, 8, and 12 h). (d) Normalized peeling strength from skin under controlled moisture exposure prior to attachment. (E) Representative images showing simultaneous 8-hour forehead wear of a conventional gel electrode and a fully encapsulated personalized kirigami patch, together with the corresponding post-removal skin condition. (F) Magnified view of the marked regions, showing visible erythema at the gel electrode site, while only minimal transient skin impressions are observed at the kirigami patch site.

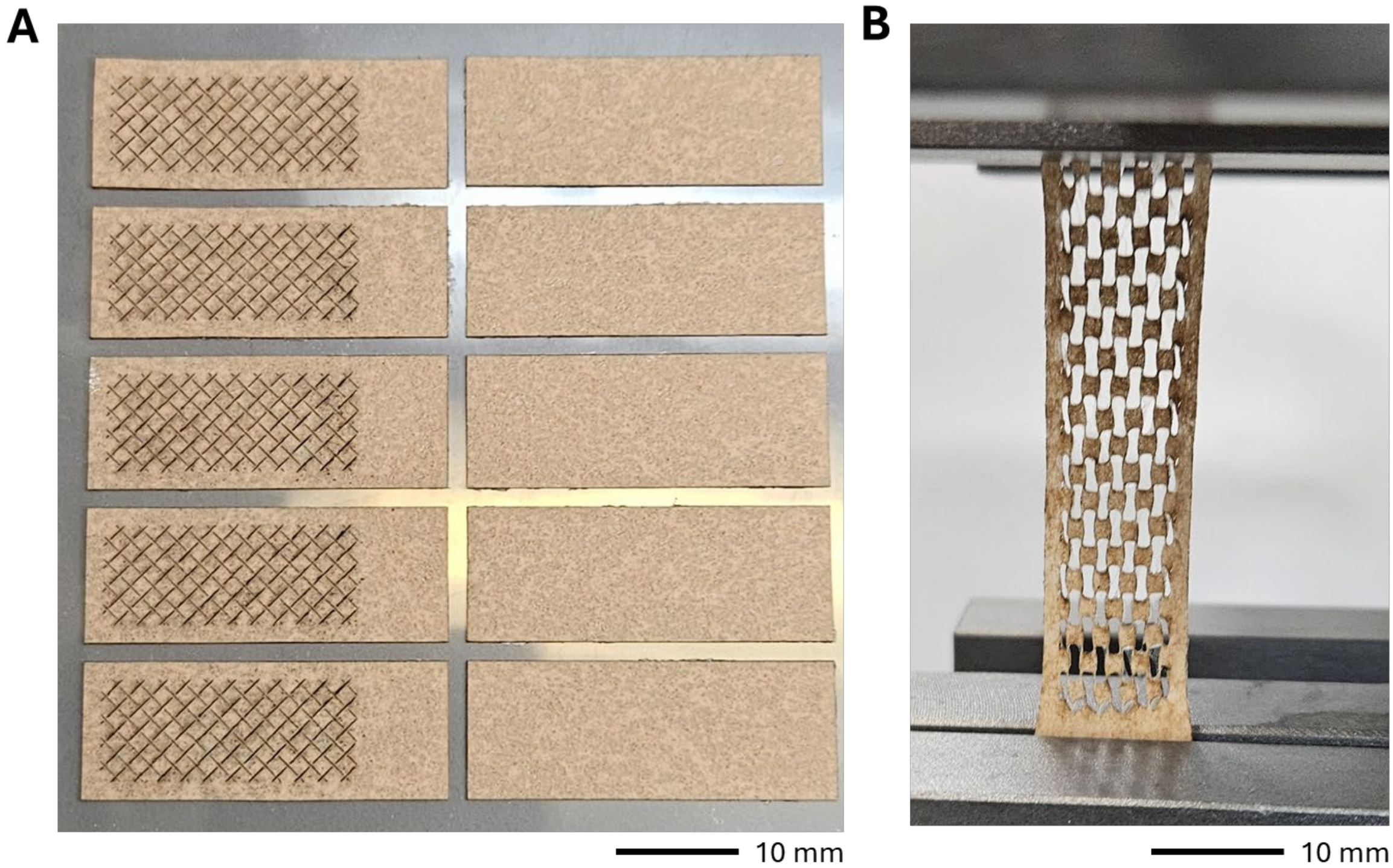


**Figure S4.** Tensile strength test (A) Optical images of fabricated plain patches and kirigami-structured patches. (B) Optical image of the kirigami patch under tensile loading, demonstrating out-of-plane deformation and structural elongation enabled by the kirigami pattern.

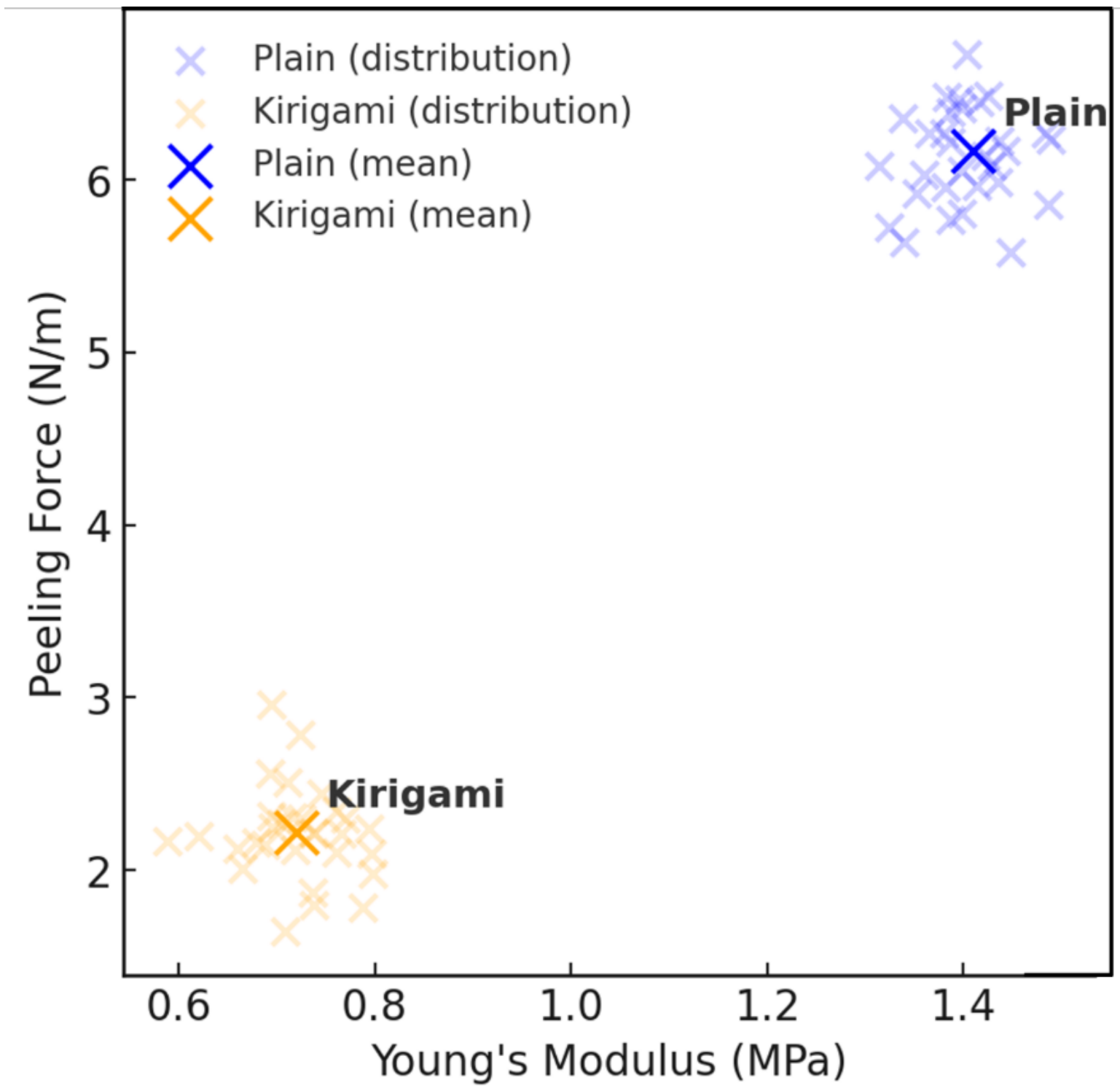


**Figure S5.** Comparison of Young's modulus and peeling force between plain and kirigami patches, demonstrating reduced stiffness and adequate skin adhesion enabled by the kirigami structure.

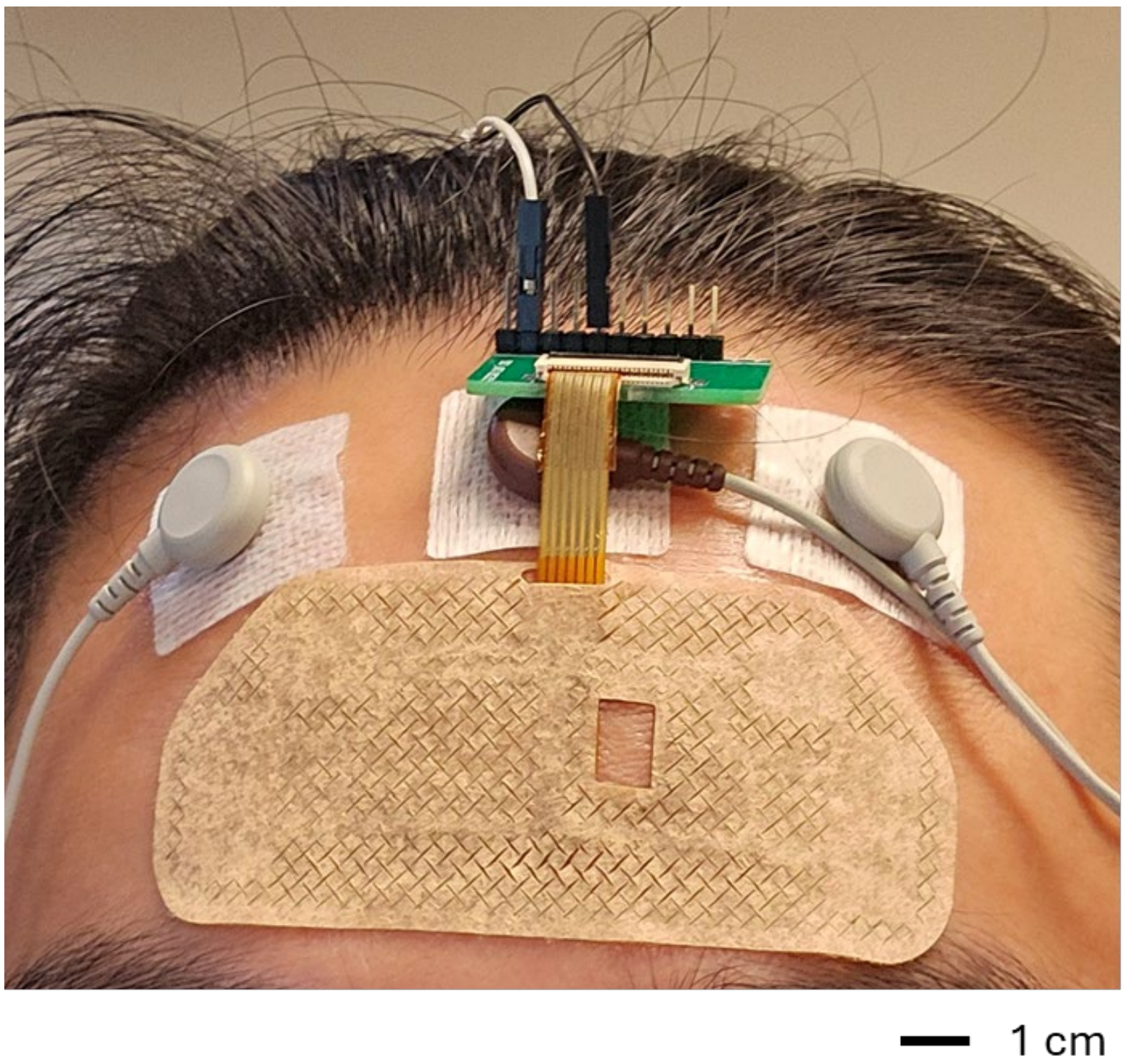


**Figure S6.** Experimental setup for simultaneous EEG acquisition using a commercial gel electrode and the kirigami patch with BioRadio.

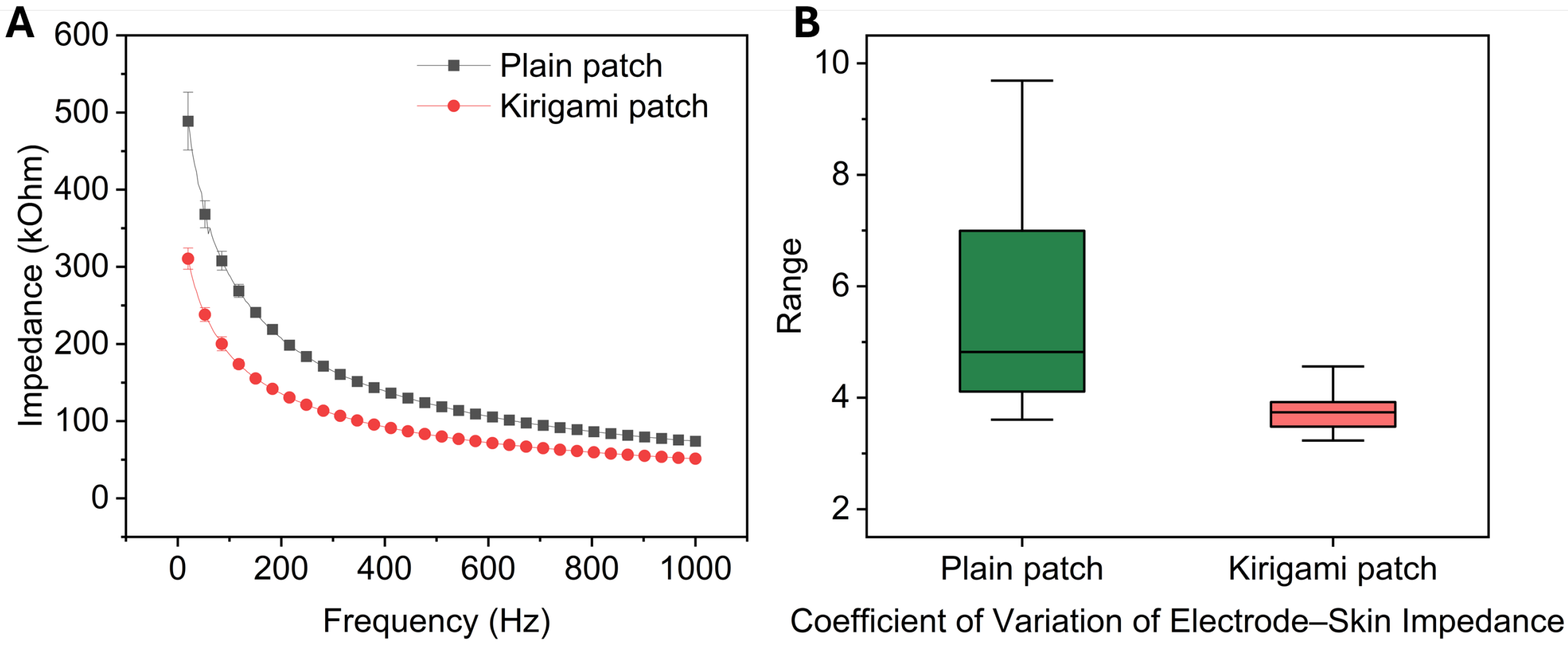


**Figure S7.** (A) Skin impedance comparison between the plain patch and the kirigami patch gold serpentine electrode on the forehead. (B) Electrode–skin impedance variation for plain and kirigami patches. Boxes show interquartile ranges, center lines indicate medians, and whiskers represent full data ranges.

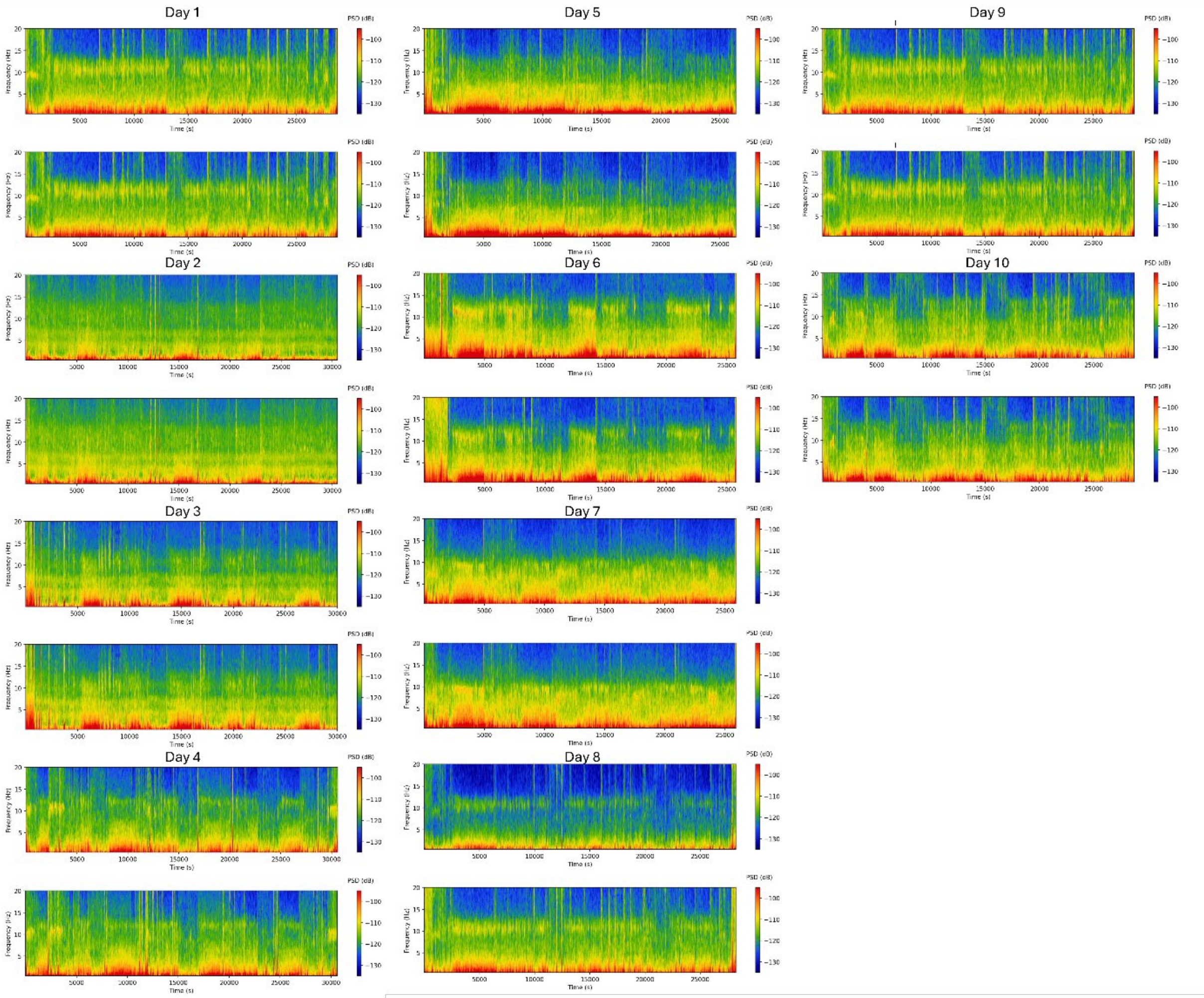


**Figure S8.** Long-term time–frequency spectrogram of overnight EEG recordings collected over 10 consecutive nights.

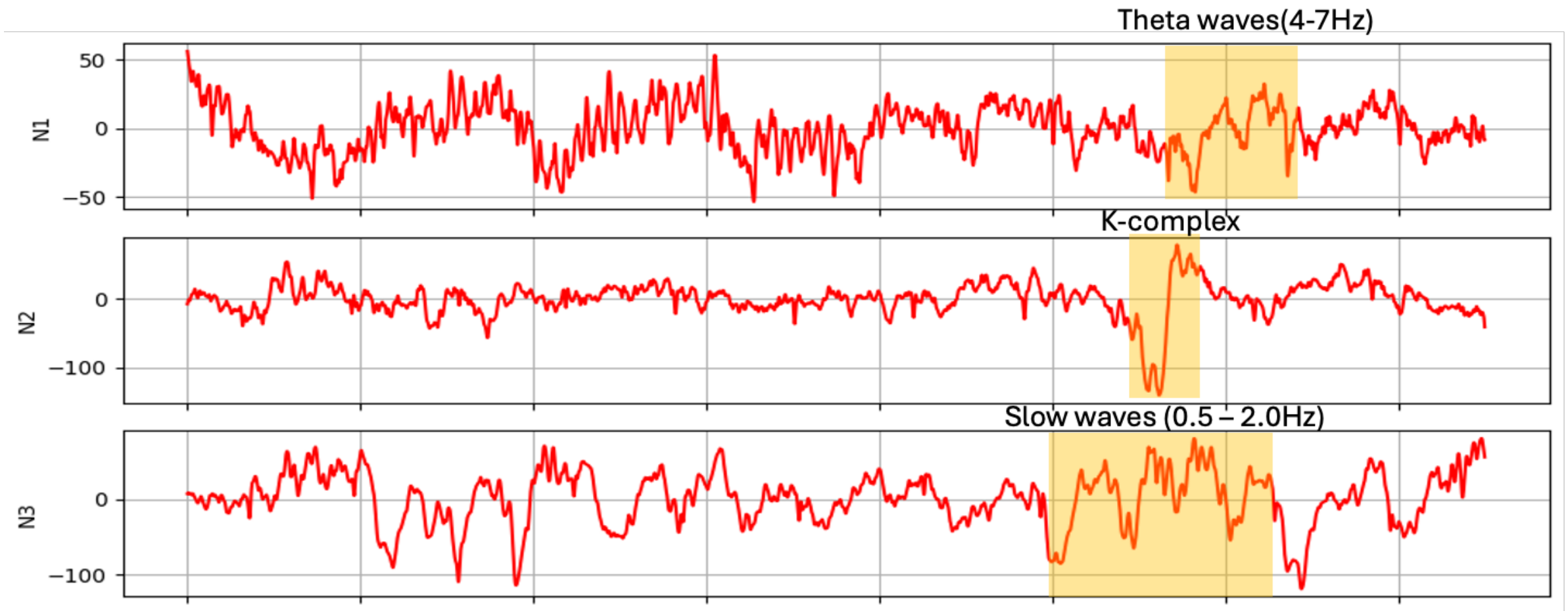


**Figure S9.** Cognitive sleep features in raw signals of each sleep stage (N1, N2, N3).

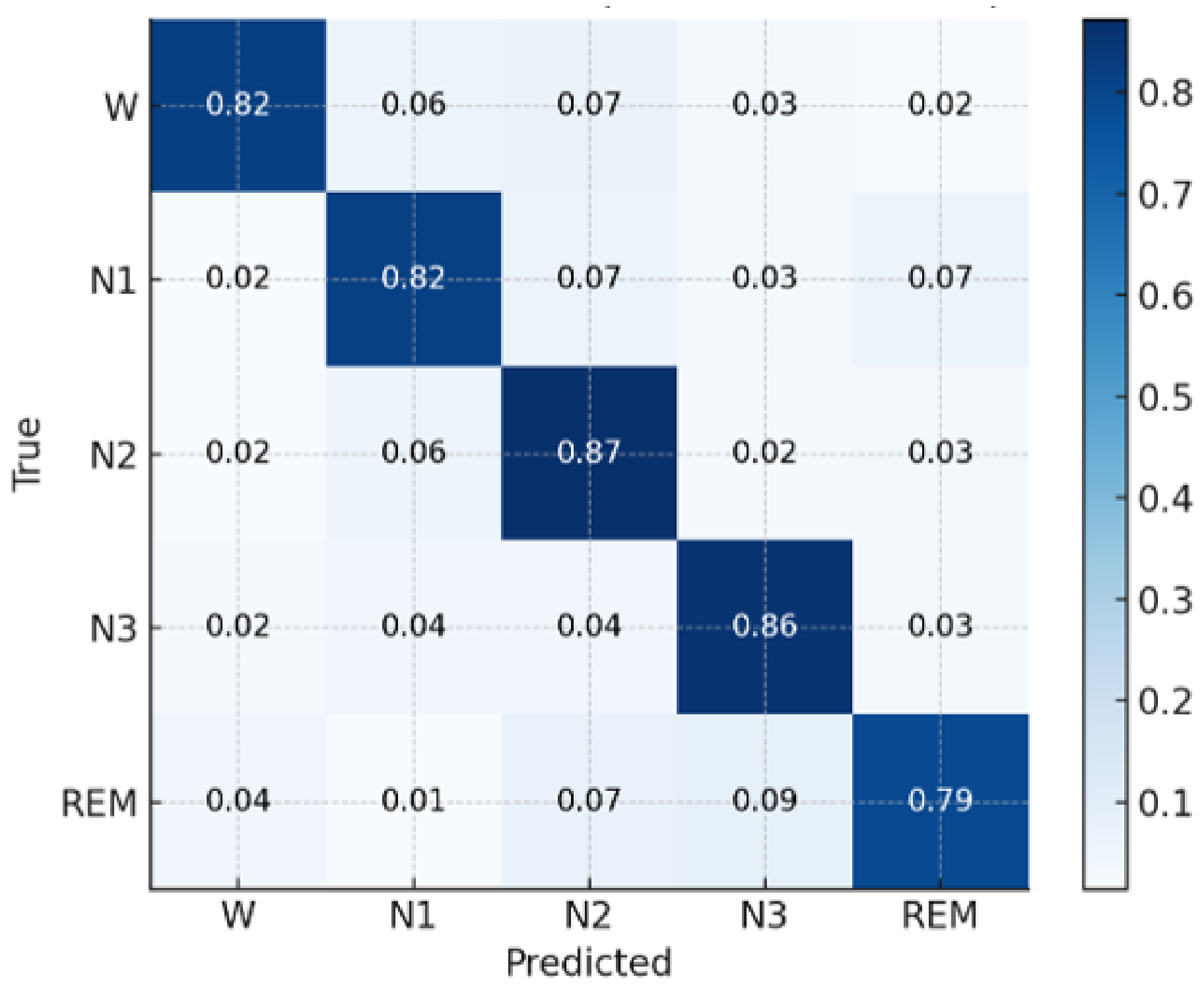


**Figure S10.** Confusion matrix of sleep stage classification performance evaluated on a publicly available dataset with expert-labeled sleep stages, used to validate the proposed sleep staging algorithm (YASA method).

**Table S1.** Design-feature-based comparison of wearable and conventional EEG monitoring approaches.

| REF | Device configuration (Device / Electrode) | Signal denoising strategy | | | | | Measured signals | SNR (dB) | Application | Electrode placement |
|---|---|---|---|---|---|---|---|---|---|---|
| | | Notch filter | Conventional filter* | Machine Learning | Morphology-adaptive | Personalization | | | | |
| This work | Patch / Serpentine electrode | O | X | X | O | O | EEG | 24.78 | Sleep | Forehead |
| 23 | Patch / Needle array | O | O | X | O | X | EEG/EOG/PPG | - | Motion / Sleep | Forehead /Arm/Leg |
| 44 | Ear buds / Silver revet | O | X | X | X | O | EEG | - | Seizure detection | Ear |
| 45 | Patch / Serpentine electrode | O | O | O | X | X | EEG/EOG/ECG | 22.77 | Sleep | Forehead |
| 46 | Patch / Hydrogel electrodes | X | O | O | O | X | EEG | - | Attention evaluation | Forehead |
| 47 | Headband / Dry electrodes | O | X | O | X | X | EEG | - | BMI | Forehead/Occipital |
| 48 | Patch / Hydrogel electrodes | O | O | X | X | X | ECG/EMG/EEG | - | - | Face |
| 49 | Patch / Hydrogel electrodes | O | X | X | X | X | EEG | 15 | Auditory | Face |
| 50 | Patch / Ambulatory electrode | O | X | - | X | X | EEG/EOG/EMG | - | Sleep | Face |
| 51 | Cap / Flexible electrode | O | O | X | X | X | EEG | 12.5 | Brain-to-brain interface | Forehead/Occipital |
| 52 | Headband / Dry electrodes | O | O | X | X | X | EEG | - | Blink-related oscillation | Forehead/Occipital |

*Conventional filter: Band-pass, time-frequency filter
